# Development of a Long-Term Sampling Method for Determination of NMHCs in Indoor Air

**DOI:** 10.3390/molecules28135001

**Published:** 2023-06-26

**Authors:** Darya Urupina, Sylvie Traverse, Thierry Leonardis, Elise Eymard-Vernain, Julien Guilhermet, Vincent Ricard, Marie Lemoine, Camille Varlet, Remy Gillet, Nadine Locoge

**Affiliations:** 1 IMT Nord Europe, Institut Mines-Telecom, Centre for Energy and Environment, University Lille, 59000 Lille, France; thierry.leonardis@imt-nord-europe.fr (T.L.); camille.varlet@imt-nord-europe.fr (C.V.); nadine.locoge@imt-nord-europe.fr (N.L.); 2 GINGER-BURGEAP, 19 Rue de la Villette, CEDEX 3, 69425 Lyon, France; s.traverse@groupeginger.com (S.T.); m.lemoine@groupeginger.com (M.L.); r.gillet@groupeginger.com (R.G.); 3 TERA Environment, 628 Rue Charles de Gaulle, 38920 Crolles, France; elise.eymardvernain@tera-environnement.com (E.E.-V.); julien.guilhermet@tera-environnement.com (J.G.); vincent.ricard@tera-environnement.com (V.R.)

**Keywords:** non-methane hydrocarbons (NMHCs), gas sampling, indoor air, vapor intrusion, method development, method validation, uncertainty calculations, breakthrough studies

## Abstract

Vapor intrusion is detrimental for indoor air quality. One of the most common sources of vapor intrusion is soil contaminated with petroleum hydrocarbons. To evaluate the long-term risk from individual exposure to hydrocarbons it is necessary to measure quantitively and reliably an average concentration level of individual pollutants on a monthly or yearly basis. Temporal variability of vapor intrusion from hydrocarbons poses a significant challenge to determination of average exposure and there is a need for reliable long-term integrative sampling. To this end, an analytical method for determination of 10 selected nonmethane hydrocarbons (NMHCs), including hexane, heptane, octane, decane, benzene, toluene, ethyl-benzene, m,p-xylene, o-xylene, and naphthalene, sampled on active triple-bed tubes filled with Carbograph 2, Carbograph 1, and Carboxen 1003 adsorbents was developed and validated. Extensive laboratory studies proved the absence of breakthrough at 50% HR and ambient temperature for experiments lasting up to 28 days and established a safe sampling time/volume of 20 days/114 L when sampling at a low flow rate of around 4 mL min^−1^. In addition, the developed method includes detailed uncertainty calculations for determination of concentrations. Finally, the method was tested by measuring NMHC concentrations in indoor air at a former industrial site during a 2-month-long field campaign in Lyon. The results of the field campaign suggest that 4-week integrated concentration measurements can be achieved by using active sampling on triple-bed tubes at 4.5 mL min^−1^.

## 1. Introduction

Indoor air quality is a major concern as, on average, people in developed countries spend 85% of their time in enclosed environments, mostly at home [1]. Vapor intrusion is a phenomenon described as infiltration of chemicals from subsurface sources, such as soil and groundwater, into an overlaying building leading to degradation of indoor air quality. Buildings especially prone to poor indoor quality due to vapor intrusion include former manufacturing facilities as well as buildings constructed on former industrial and waste-disposal sites.

In the presence of soil contamination, indoor air sampling is an important step for evaluation of intrusion of hydrocarbons into the overlaying building and thus providing risk assessment for occupants. Multiple methods exist for long-term monitoring of non-methane hydrocarbons (NMHCs), such as passive sampling, online sampling, collection into sampling tubes, or collection of whole air in canisters or other receptacles [2,3]. These approaches to indoor air sampling can be roughly divided into three types, each presenting its own challenges: (i) online continuous monitoring, (ii) 1–2-week passive sampling and (iii) multiple rounds of short-time active sampling. Online continuous monitoring of pollutants by direct automated methods is robust and reliable but is much too expensive and cumbersome for routine monitoring of indoor pollution. Passive sampling is well adapted for up to two-week sampling, but relies on correct values of the analytes’ uptake rates, which is linked to environmental conditions (humidity, temperature, pollutant concentration) and experimental parameters (sampling time, presence of co-pollutants, variability of concentration). Depending on the analytes, changes in these parameters can introduce important uncertainty into the measurements. Nevertheless, for many pollutants, such as benzene, passive sampling is very robust [4]. In France, one-week measurement using radial passive samplers is a preferred means of investigation of indoor pollution. Long-term risk for individual exposure to hydrocarbons can also be evaluated by multiple rounds of short-time active sampling to take into account temporal variability [5]. Active sampling does not depend on the diffusion rates, and the calculated concentration is only a function of the sampled volume as long as sampling is performed within the sorbent adsorption capacity. In the USA, active sampling using Tenax TA-filled sorbent tubes followed by thermal desorption is by far the most prevalent method for determination of benzene in ambient air [2]. For example, to measure the average annual concentration of a number of volatile organic compounds (VOCs) including benzene, toluene, ethylbenzene, and xylenes (BTEX), Baya et al. collected one duplicate sample every month by sampling for 1 h at 100 mL min^−1^ using stainless steel sampling tubes filled with 500 mg of Tenax TA [6]. This approach risks missing periods of high or low concentrations and can therefore underestimate or overestimate overall exposure.

To better evaluate the long-term risk for individual exposure to hydrocarbons it is necessary to quantitively and reliably measure an average concentration level of the pollutants in a building on a monthly or yearly basis. Temporal variability of VOC concentrations should also be taken into consideration, as recommended by regulatory agencies and professionals working in the field [5,7,8,9,10,11]. Thus, to account for temporal annual variability in VOC concentrations it is desirable to be able to sample continuously for a longer period of time, such as biweekly or monthly. To this end, a long-term sampling method for determination of chosen hydrocarbon pollutants using active tubes followed by thermal desorption would represent a reliable, robust, and cost-effective method for concentration measurement. In accordance with the European norms ISO 16017-1 for sampling of VOCs in indoor air by sorbent tubes followed by thermal desorption and analysis by gas chromatography, the upper recommended sampling time can be stretched to 33 h if sampling the recommended maximum volume of 10 L at the minimum recommended rate of 5 mL min^−1^ [12]. Longer sampling periods can be adopted by using novel, more capacitive tubes. Nevertheless, it is important to keep in mind that sampling in the order of weeks will expose the tube sorbent to high amounts of water which can lead to earlier breakthrough of the measured compounds. Hence, choosing a suitable type of active sampling support and defining the maximum sampling period is absolutely crucial.

The goal of this work is to develop a practical cost-effective 7-day- to 1-month-long active sampling method using low-flow pumps and multi-bed sorbent sampling tubes compatible with thermal desorption for determination of indoor hydrocarbon pollutants containing from 6 to 10 atoms of carbon (C6–C10), such as hexane, heptane, octane, decane, benzene, toluene, ethyl-benzene, m,p-xylene, o-xylene, and naphthalene. To this end, the following strategy was adopted: (i) develop an analytical method for determination of 10 selected NMHCs actively sampled on triple-bed tubes for extensive periods of time up to 28 days; (ii) prove that breakthrough volume is not achieved during long-time analysis or alternatively define the breakthrough volume for compounds demonstrating breakthrough behavior; (iii) calculate the uncertainty of the method for concentration determination, including both volume and mass uncertainties; (iv) test the method during a 2-month-long campaign.

## 2. Results and Discussion

### 2.1. Method Validation

A GC-MS method for determination of selected hydrocarbons in triple-bed tubes employing thermal desorption was developed by TERA Environment and is described in detail in the Section 3.4. Ideally, direct liquid injection of analytes would be used to create a calibration curve and validate the analytical method for linearity within the range and repeatability of the analytical system. Due to technical configuration, the above-mentioned option was not possible, and widely-used Tenax tubes were employed instead for part of the validation studies, specifically (i) linearity and range and (ii) repeatability. Tenax tubes were doped with analytes of interest by vaporizing the liquid in a flow of helium. LOQ and LOD of the analytical method were determined using empty stainless steel tubes. The sampling performance of TB tubes was validated for accuracy, reproducibility, and stability. Masses of analytes in the unexposed TB tubes were likewise determined.

#### 2.1.1. Validation of Analytical System

Limit of detection (LOD) and limit of quantification (LOQ) were obtained by using six unexposed empty tubes as blanks. The limit of detection was established as three standard deviations of the signal of the blanks. The limit of detection was calculated as 10 standard deviations of the blank plus average mass of 6 blanks. The results are listed in Table 1. LOQ was below 1 ng for all the analytes except toluene for which LOQ was 1.60 ng.

Linearity was investigated in two ranges: from 5 to 200 ng and from 500 to 10,000 ng (for m,p-xylene linearity was investigated from 10 to 400 ng and from 1000 to 20,000 ng). For the low range, Tenax tubes were doped with 5, 20, 40, 100, and 200 ng of each NMHC using a gaseous doping system (linearity for the mixture of m,p-xylene was investigated by doping at 10, 40, 80, 200, 400 ng), and a plot of the peak area vs. theoretical mass was constructed. Using the obtained plot, 6 linear regression curves were obtained for each of the 10 analytes and the resulted coefficients of determination (R^2^) were consistently evaluated at R^2^ ≥ 0.99 proving linearity (Table 2). For the high range, Tenax tubes were doped with 500, 1000, 2000, 5000, and 10,000 ng of each NMHC (1000, 2000, 4000, 10,000, and 20,000 ng for m,p-xylene) and the procedure was analogous to the one described for the low level. The results prove a linear response of the analytical system in the defined low and high ranges for all analytes (Table 2).

Repeatability was tested to measure the sensitivity of the analytical system towards the errors coming from the instrument itself: the thermal desorber, the column, the detector, and the integration device. Tenax tubes were doped 6 times on the same day by the same analyst at each concentration level; analysis was run using the same analytical system and repeatability was evaluated as percent standard deviation (%RSD) of 6 measured mass values. The results of the study are listed in Table 3. Note that m,p-xylene was tested at a higher level than other analytes as it contains two isomers of xylene: meta- and paraforms.

#### 2.1.2. Validation of Sampling Support

Blanks/masses of the selected NMHCs were evaluated in four blanks of TB tubes and the average of four assays is listed in Table 4. While most analytes were not detected in the TB tubes above LOD level, benzene was an exception and was detected at the amount of 1.0 ng. However, the level of benzene did not exceed the LOQ and can be ignored.

Accuracy of the entire method including adsorption/desorption on the “novel” TB tubes was evaluated as %recovery of the doped analytes. Six TB tubes were doped on six different days at each mass level. Using a freshly prepared calibration curve, obtained using Tenax tubes, masses at each point were obtained and an average mass for each level was calculated. Percent recovery was then calculated as a ratio of average mass of six injections over theoretical value (Equation (1), Table 5). The method was found to be accurate with the %recovery ranging from 70 to 135%.

(1) $$Recovery=(\frac{Average\;mass\;from\;6\;injections}{Theoretical\;value})\times 100$$

Reproducibility of the entire method including adsorption/desorption on TB tubes was calculated as %RSD of six measured mass values obtained from doping six TB tubes on six different days at each level of mass (Table 6). Note that m,p-xylene was tested at a higher level than other analytes as it contains two isomers of xylene: meta- and paraforms. Using the linear calibration curve constructed by doping Tenax tubes, the amount of analytes in doped TB tubes was calculated. Reproducibility was found to be below 20% for levels of 20 ng and above. Reproducibility was less than 25% for the level of 5 ng.

Stability of the method was evaluated by doping 9 TB tubes at the highest levels of the low (5 ng to 200 ng) and high (500 ng to 10,000 ng) ranges of masses on the tube and storing 3 of these tubes at ambient temperature for 21 days. Stability was evaluated by calculating the ratio of average mass detected in 3 tubes that were analyzed 21 days after doping over average mass detected in 6 tubes analyzed immediately after doping (Equation (2), Table 7).

(2) $$Stability=(\frac{Average\;mass\;in\;3\;tubes\;after\;21\;days\;of\;storage}{average\;mass\;of\;6\;tubes\;analysed\;immediately})$$

Note that m,p-xylene was tested at higher levels than other analytes as it contains two isomers of xylene: meta- and para- forms. The masses of 10 selected NMHCs stored for 21 days were between 80 and 120% of the average masses detected in the tubes analyzed immediately (Table 7). Results indicate the stability of the analytes in the TB sampling tubes for up to 21 days for all analytes.

In conclusion, the developed analytical method was successfully validated for linearity, range, and repeatability using Tenax tubes. The validated range was established from 5 ng to 10,000 ng for hexane, heptane, octane, decane, benzene, toluene, ethyl-benzene, m,p-xylene, o-xylene, and naphthalene. The validated range for m,p-xylene is from 10 to 20,000 ng. LOQ and LOD of the system were established using empty tubes. The use of TB sampling tubes was validated by investigating the accuracy and reproducibility of adsorption/desorption and establishing the stability of sampled analytes for up to 21 days.

### 2.2. Uncertainty Calculations

Uncertainly calculations were performed following Afnor ISO 22065 and 14662-1 guidelines. These calculations were carried out on the assumption of 100% sampling efficiency of sampling support and thus uncertainty in sampling efficiency equal to zero, meaning that all the analytes that pass through the tube are adsorbed by the sampling support. This assumption is proven by breakthrough studies (Section 2.3).

Concentration of an analyte in the air is calculated using Equation (3):

(3) $$\frac{u^{2}\left(C_{m}\right)}{C_{m}^{2}}=\frac{u^{2}\left(V_{sam,STP}\right)}{V_{sam,STP}^{2}}+\frac{u^{2}\left(m_{sam}\right)+u^{2}\left(m_{bl}\right)}{{\left(m_{sam}-m_{bl}\right)}^{2}}$$

where $C_{m}$ is the measured concentration in µg m^−3^, ${u(C}_{m})$ is the uncertainty of the measured concentration in µg m^−3^, $V_{sam,STP}$ is the sampled volume under STP conditions in L, ${u(V}_{sam,STP})$ is the uncertainty of the sampled volume under STP conditions in L, $m_{sam}$ is the mass of the analyte sampled in the tube in ng, ${u(m}_{sam})$ is the uncertainty of the mass of the analyte sampled in the tube in ng, $m_{bl}$ is the mass of the analyte in the blanks of the sampling tubes in ng, $u(m_{bl})$ is the uncertainty of the mass of the analyte in the blanks of the sampling tubes in ng.

For volume-controlled sampling devices, the following conversion can be used to calculated volume at STP (Equation (4)):

(4) $$V_{sam,STP}=V_{sam}\frac{\bar{P}}{101.3}\frac{293}{\left(\bar{T}\right)}$$

where $V_{sam}$ is the actual measured volume in L, $\bar{P}$ is the average air pressure during the sampling in kPa, and $\bar{T}$ is the average air temperature during sampling in K.

If the mass of the analyte in the blanks is below the LOQ of the analytical system, then the formula can be simplified to Equation (5):

(5) $$\frac{u^{2}\left(C_{m}\right)}{C_{m}^{2}}=\frac{u^{2}\left(V_{sam,STP}\right)}{V_{sam,STP}^{2}}+\frac{u^{2}\left(m_{sam}\right)}{{\left(m_{sam}\right)}^{2}}$$

Thus, combined relative concentration uncertainty represented by the term $\frac{u^{2}\left(C_{m}\right)}{C_{m}^{2}}$ is the sum of relative volume uncertainty at STP $\frac{u^{2}\left(V_{sam,STP}\right)}{V_{sam,STP}^{2}}$ and relative sampled mass uncertainty $\frac{u^{2}\left(m_{sam}\right)}{{\left(m_{sam}\right)}^{2}}$. Calculations of mass uncertainty were made using validation data provided by TERA Environment, presented in the previous section. Volume uncertainty was calculated following 14662-1 guidelines while conducting breakthrough experiments by measuring flow rate at the beginning and at the end of each breakthrough experiment, and is discussed later.

#### 2.2.1. Mass Uncertainty

Mass uncertainty for low and high levels of concentration was calculated based on data provided by TERA Environment according to the norms Afnor ISO 22065 and 14662-1 [13,14] (Equation (6)):

(6) $$\frac{u^{2}\left(m_{sam}\right)}{m_{sam}^{2}}=\frac{u^{2}\left(m_{meas}\right)}{m_{meas}^{2}}+\frac{u^{2}\left(D\right)}{D^{2}}+U_{st}^{2}$$

where $\frac{u(m_{meas})}{m_{meas}}$ is the relative uncertainty related to the mass measurement by the analytical system, $\frac{u\left(D\right)}{D}$ is the relative uncertainty related to adsorption/desorption, and $U_{st}$ is the relative uncertainty related to the storage of the tubes. Relative uncertainty of the mass measurement by the analytical system $\frac{u(m_{meas})}{m_{meas}}$ is calculated as follows (Equation (7)):

(7) $$\frac{u^{2}\left(m_{meas}\right)}{m_{meas}^{2}}=\frac{u^{2}\left(m_{cs}\right)}{nm_{cs}^{2}}+w_{F}^{2}+w_{d}^{2}{+w}_{anal}^{2}$$

where $\frac{u(m_{cs})}{m_{cs}}$ is the relative uncertainty of the mass in the corresponding calibration standards, n is the number of calibration standards used to construct the calibration function, $w_{F}$ is random uncertainty due to calibration (lack-of-fit of the calibration function) and is estimated by TERA Environment at 2%, $w_{d}$ is systematic uncertainty due to drift in the equipment signal between two consecutive calibrations and is ignored due to performance of frequent calibrations (every 15 samples), $w_{anal}$ is random uncertainty related to the repeatability of the analytical method. Uncertainty due to repeatability of the analytical method $w_{anal}$ was calculated as the RSD of 6 analyses of 10 selected NMHCs on Tenax tubes under the conditions of repeatability for 10 mass levels, presented above in Table 3.

Relative uncertainty of mass in reference solutions used for calibration $\frac{u(m_{cs})}{m_{cs}}$ is the sum of purity uncertainty of reference solutions $u_{m}$ (provided by the Restek vendor at 1%), a term related to the accuracy of the micropipette/syringe used to prepare reference solutions $N\frac{{\left(B_{max,sy,ref}\right)}^{2}}{3}$, and a term related the precision of the micropipette/syringe used to prepare reference solutions $N\frac{{\left(K_{v,sy,ref}\right)}^{2}}{3}$ (Equation (8)).

(8) $$\frac{u^{2}\left(m_{cs}\right)}{nm_{cs}^{2}}={\left(u_{m}\right)}^{2}+N\frac{{\left(B_{max,sy,ref}\right)}^{2}}{3}+N\frac{{\left(K_{v,sy,ref}\right)}^{2}}{3}$$

$B_{max,sy,ref}$ is the maximum tolerated bias between a measured value and a true value according to the micropipette used to deliver the volume, estimated by the provider at 2%, $K_{v,sy,ref}$ is the maximum tolerated repeatability of the micropipette, estimated by the provider at 1%, and N is the number of dilutions necessary to obtain the required concentration of the reference solution. The terms $B_{max,sy,ref}$ and $K_{v,sy,ref}$ are divided by $\sqrt{3}$ as uncertainty is evaluated using rectangular probability density function.

Relative uncertainty related to adsorption/desorption $u\left(D\right)$ is calculated according to Equation (9):

(9) $$u^{2}\left(D\right)=\frac{({B_{a})}^{2}}{3}+\frac{{{(K}_{v,ra})}^{2}}{n}+{\left(u_{ks}\right)}^{2}$$

where $\frac{({B_{a})}^{2}}{3}$ is the uncertainty term related to accuracy of adsorption/desorption on TB tubes, $\frac{{{(K}_{v,ra})}^{2}}{n}$ is the uncertainty term related to the reproducibility of adsorption/desorption on TB tubes, ${\left(u_{ks}\right)}^{2}$ is the uncertainty term related to the purity of the solutions used for doping the TB tubes, and n is the number of assays used to evaluate recovery (in this case *n* = 6).

More specifically, $B_{a}$ is the bias of doping (Equation (10)):

(10) $$B_{a}=|1-\frac{average\;of\;6\;masses\;measured\;after\;doping\;tubes\;at\;the\;defined\;theoretical\;mass\;level}{defined\;theoretical\;mass}|$$

where bias of recoveries for 10 analytes based on 6 measurements per each mass level was obtained during accuracy studies and is listed in Table 8. $B_{a}$ is divided by $\sqrt{3}$ as uncertainty is evaluated using rectangular probability density function.

Uncertainty related to reproducibility of measurements due to adsorption/desorption on TB tubes was determined experimentally and calculated in the following manner (Equation (11)):

(11) $${(K}_{v,ra})=RSD\;of\;6\;mass\;values\;measured\;after\;doping\;tubes\;at\;the\;defined\;theoretical\;level$$

where reproducibility of doping for 10 analytes defined as RSD can be obtained from Table 6. In Equation (9), ${(K}_{v,ra})$ is divided by $\sqrt{6}$ as 6 assays were done to obtain %RSD of mass values for each mass level and a Gaussian distribution function is appropriate when a set of values is randomly distributed.

The uncertainty term related to purity of the solutions used for doping the TB tubes $u_{ks}$ is calculated using Equation (12):

(12) $${\left(u_{ks}\right)}^{2}={\left(u_{purity}\right)}^{2}+N\frac{{\left(B_{max,sy,doping}\right)}^{2}}{3}+N\frac{{\left(K_{v,sy,doping}\right)}^{2}}{3}$$

where $u_{purity}$ is the uncertainty of the doping solution, provided by the vendor at 0.006, $N\frac{{\left(B_{max,sy,doping}\right)}^{2}}{3}$ is a term related to the accuracy of the micropipette used to prepare doping solutions, and $N\frac{{\left(K_{v,sy,doping}\right)}^{2}}{3}$ is a term related the precision of the micropipette used to prepare doping solutions. $B_{max,sy,doping}$ is the maximum tolerated bias of the accuracy of the micropipette used to deliver the volume, estimated by the provider at 0.02, $K_{v,sy,doping}$ is the maximum tolerated value for repeatability of the micropipette used to deliver the volume, estimated by the provider at 0.01, and N is the number of dilutions necessary to obtain the required concentration of doping solution. $B_{max,sy,doping}$ and $K_{v,sy,doping}$ are both maximum tolerated values and they are divided by $\sqrt{3}$ as uncertainty is evaluated using rectangular probability density function.

Finally, the uncertainty due to stability of the analyte during storage is evaluated using Equation (13):

(13) $$U_{st}=\frac{\Delta st}{\sqrt{3}}$$

where Δst=(1−recovery at 21 days). Recoveries after 21 days of ageing can be found in Table 7. Recoveries at 200 ng were used to calculate stability of NMHCs for levels from 5 to 200 ng, while recoveries at 10,000 ng were used to calculate stability of NMHCs for levels from 500 to 10,000 ng (for m,p-xylene recovery at 400 ng was used to calculate stability for levels from 10 to 400 ng, while recoveries at 20,000 ng were used to calculate stability of NMHCs for levels from 1000 to 20,000 ng). Uncertainty is evaluated using rectangular probability density function and thus Δst is divided by $\sqrt{3}$.

Table 9 summarizes standard mass uncertainties for the measurement of 10 NMHCs at each level of mass. They vary from 7% to 26%. The upper limit of 26% was obtained for naphthalene at the lowest level of mass (5 ng) and was due to poor recovery on TB tubes at this level. As discussed earlier, standard mass uncertainty is the sum of uncertainties related to the mass measurement by the analytical system ($u(m_{mes})$), adsorption/desorption ($u\left(D\right)$), and stability ($U_{st}$). Uncertainty related to the mass measurement by the analytical system $u(m_{mes})$ for all levels of mass and for all compounds ranged from 4% to 14%, with 95% of the results ranging between 5% and 10%. This represents a very stable contribution to the overall uncertainty. Uncertainty in the measurements of mass due to stability $U_{st}$ investigated at 200 ng and at 10,000 ng (400 ng and 20,000 ng for m,p-xylene) was also rather low, ranging from 1% to 10%. The uncertainty related to adsorption/desorption,$u\left(D\right)$, at ten levels of mass showed higher variation of values and ranges between 3% to 23%. These variations were random and did not show a clear dependence on mass levels. Generally speaking, uncertainty of mass measurements (over 20%) is associated with high uncertainty due to adsorption/desorption (over 15%) which in its turn can be traced to instances of poor recovery on TB tubes. Variations in overall standard mass uncertainties were also random, mostly triggered by random variations of uncertainty due to adsorption/desorption, and no clear pattern of increase or decrease of uncertainty with measured mass was observed. Thus, a practical way to assign uncertainty to the results is to calculate an average uncertainty for low and high levels of mass, excluding the uncertainty at 5 ng because multiple compounds, namely hexane, benzene, and naphthalene, have unusually high contributions to the uncertainty at this level due to high adsorption/desorption uncertainty. Table 10 summarizes the practical mass uncertainty at 67% and 95% confidence levels for three ranges of mass: <20 ng, 20–200 ng, and 201–10,000 ng (in the case of m,p-xylene: <20 ng, 20–400 ng, and 401–20,000 ng). Expanded uncertainty at 95% confidence level is obtained by multiplying $\frac{u\left(m_{sam}\right)}{m_{sam}}$ by 2.

#### 2.2.2. Volume Uncertainty

Volume uncertainty was calculated from laboratory studies data aimed at exploring the breakthrough volume of 10 NMHCs of interest in accordance with the norm 14662-1 [14]. The following formula expresses the volume uncertainty $\frac{u^{2}\left(V_{sample,STP}\right)}{V_{sample,STP}^{2}}$ at STP as the sum of uncertainties related to (i) the act of measurement of the flow of the pump using a flow meter $\frac{u^{2}\left(\varphi_{start}\right)+u^{2}\left(\varphi_{end}\right)}{{\left(\varphi_{start}+\varphi_{end}\right)}^{2}}$, (ii) variation of the flow during experiment $\frac{\Delta^{2}\varphi }{12({\frac{\varphi_{start}+\varphi_{end}}{2})}^{2}}$, and (iii) measurement of time $\frac{u^{2}\left(t\right)}{t^{2}}$, (iv) temperature $\frac{u^{2}\left(\bar{T}\right)}{{\bar{T}}^{2}}$, and (v) pressure $\frac{u^{2}\left(\bar{P}\right)}{{\bar{P}}^{2}}$ during the experiment. Thus, using Equation (14):

(14) $$\frac{u^{2}\left(V_{sample,STP}\right)}{V_{sample,STP}^{2}}=\frac{u^{2}\left(\varphi_{start}\right)+u^{2}\left(\varphi_{end}\right)}{{\left(\varphi_{start}+\varphi_{end}\right)}^{2}}+\frac{\Delta^{2}\varphi }{12({\frac{\varphi_{start}+\varphi_{end}}{2})}^{2}}+\frac{u^{2}\left(t\right)}{t^{2}}+\frac{u^{2}\left(\bar{T}\right)}{{\bar{T}}^{2}}+\frac{u^{2}\left(\bar{P}\right)}{{\bar{P}}^{2}}$$

where $\varphi_{start}$ is the measured flow of the pump in ml min^−1^ before sampling based on three consecutive measurements, $\varphi_{end}$ is the measured flow of the pump in ml min^−1^ after sampling based on three consecutive measurements, $u(\varphi_{start})$ and $u(\varphi_{end})$ are the standard uncertainties in the measurement of the flow in ml min^−1^ at the beginning and at the end of the experiment, respectively, $\Delta \varphi =\varphi_{start}-\varphi_{end}$ is the difference in the flow between the beginning and the end of the experiment in ml min^−1^, u(t) is the standard uncertainty related to the measurement of time of the experiment, here defined as 0.5 min, $u(\bar{T})$ is the uncertainty related to the variation of temperature during the experiment in K, $u\left(\bar{P}\right)$ is the uncertainty related to the variation of pressure during the experiment in kPa, and t is the overall sampling time of the experiment in min.

The uncertainty term related to the act of measurement of the flow of the pump using a flow meter $\frac{u^{2}\left(\varphi_{start}\right)+u^{2}\left(\varphi_{end}\right)}{{\left(\varphi_{start}+\varphi_{end}\right)}^{2}}$ requires calculation of uncertainties of flow measurement at the beginning $u\left(\varphi_{start}\right)$ and at the end $u(\varphi_{end})$ of the experiment. Below is the calculation of uncertainty in the measurement at the beginning of the experiment. Uncertainty in the flow measurement at the end of the experiment is calculated analogously using Equation (15):

(15) $$\frac{u^{2}\left(\varphi_{start}\right)}{{{(\varphi }_{start})}^{2}}=\frac{{\left(u_{cal}\right)}^{2}+\frac{s_{meas}^{2}}{n}}{{{(\varphi }_{start})}^{2}}+\frac{u^{2}\left(T_{start}\right)}{{T_{start}}^{2}}+\frac{u^{2}\left(P_{start}\right)}{{P_{start}}^{2}}$$

where $u_{cal}$ is the uncertainty related to the calibration of a flow meter in ml min^−1^, $s_{meas}$ is the standard deviation of n successive measurements (minimum 3) while determining average gas flow $\varphi_{start}$ at the beginning of the experiment in ml min^−1^, $u(T_{start})$ is the uncertainty related to the measurement of temperature by the flow meter during measurement of the flow at the beginning of the experiment in K, $u\left(P_{start}\right)$ is the uncertainty related to the measurement of pressure by the flow meter during measurement of the flow at the beginning of the experiment in kPa, $T_{start}$ is the temperature when measuring $\varphi_{start}$ in K, and $P_{start}$ is the pressure when measuring $\varphi_{start}$ in kPa. The uncertainties $u_{cal}$, $u(T_{start})$, and $u\left(P_{start}\right)$ are calculated using relative uncertainties of the flow meter DRYCAL 0–500 related to measurement of flow, temperature, and pressure, respectively, at a 75% confidence level provided by the manufacturer. Thus:

$$u_{cal}=0.0007{(\varphi }_{start})$$

$$u(T_{start})=0.02{(\varphi }_{start})$$

$$u\left(P_{start}\right)=0.0003{(\varphi }_{start})$$

The uncertainties related to the variation of temperature and pressure for the whole duration of the experiment were calculated from maximum and minimum values for T and P during the experiment, assuming their even distribution (Equations (16) and (17)).

(16) $$u\left(T\right)=u_{cal\;therm}+\frac{{\left(T_{max}-T_{min}\right)}^{2}}{12}$$

where $u_{cal\;therm}$ in K is the uncertainty due to calibration of the thermometer, too small in comparison with the second term and therefore ignored.

Similarly:

(17) $$u\left(P\right)=\frac{{\left(P_{max}-P_{min}\right)}^{2}}{12}$$

Table 11 lists the individual volume uncertainties for 80 runs. Note that in three cases a pump stopped during the experiment and the results were discarded. This happened during long-term experiments of 14 days and longer. Moreover, the chance of a complete stop of a sampling pump or large fluctuation of flow measured at the beginning and the end of the experiment increased with time of use of a pump, due to wearing of its membrane with prolonged continuous use. This can be a major inconvenience, as maintenance considerably increases the total cost of implementing active long-term sampling using sampling pumps. For the rest of the results, volume uncertainty was generally low, below 2.5% for 96% of the results. If we look closer at what caused uncertainty higher than 2.5%, it was always linked to higher fluctuation of flow during the experiment. Thus, uncertainty of 2.81% measured during 3-day sampling was linked to an increase in the flow of 9%, uncertainty of 2.69% obtained during 21-day sampling was linked to a 9% increase in the flow, and uncertainty of 6.91% was linked to a decrease in the flow of 27%. Uncertainty due to the measurement of the time of the experiment t ranged from 10^−8^ for 8 h experiments to 10^−12^ for 28-day experiments and can be neglected.

A practical way to use the results of the “volume uncertainty” study is to apply 2.5% volume uncertainty (5% expanded volume uncertainty) to all the results of future investigations carried out with the same type of sampling pumps, provided that the fluctuation of the flow between the beginning and the end of the experiments does not exceed 8%. Thus, the maximum tolerated value of flow variation was established at 8% and the results of the experiments exceeding this value were rejected.

#### 2.2.3. Concentration Uncertainty

Concentration uncertainty was calculated as the sum of volume uncertainty and mass uncertainty, as expressed in Equation (18):

(18) $$\frac{u\left(C_{m}\right)}{C_{m}}=\sqrt{\frac{u^{2}\left(V_{sam,STP}\right)}{V_{sam,STP}^{2}}+\frac{u^{2}\left(m_{sam}\right)}{{\left(m_{sam}\right)}^{2}}}$$

As mass uncertainties in our studies were much larger than volume uncertainty defined at 2.5%, the mass uncertainty term dominates the calculations of concentration uncertainty. A maximum increase of 1% was observed when comparing mass uncertainty to concentration uncertainty. Table 12 summarizes standard concentration uncertainties for the measurement of 10 NMHCs at each level. They vary from 8% to 26%.

Expanded concentration uncertainty at 95% confidence level is obtained by multiplying $\frac{u\left(C_{m}\right)}{C_{m}}$ by 2. Similar to the practical standard mass uncertainties, practical standard concentration uncertainties were obtained by calculating average practical concentration uncertainties for three ranges of concentrations: <20 ng, 20–200 ng, and 201–10,000 ng (in the case of m,p-xylene: <20 ng, 20–400 ng, and 401–20,000 ng). Practical expanded concentration uncertainties were obtained in an analogous way. The results are listed in Table 13.

### 2.3. Breakthrough Studies

**Figure 1:**
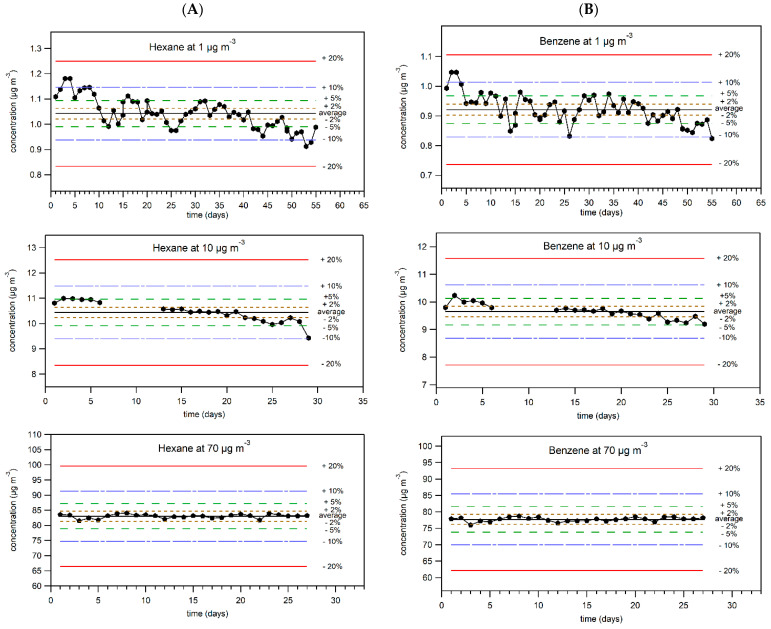
Stability of generated concentrations of (**A**) hexane and (**B**) benzene in breakthrough studies. In most cases, each point represents an average of 24 measurements. Sometimes, an average of 5 to 23 measurements was taken, depending on availability of analytical data.

**Figure 2:**
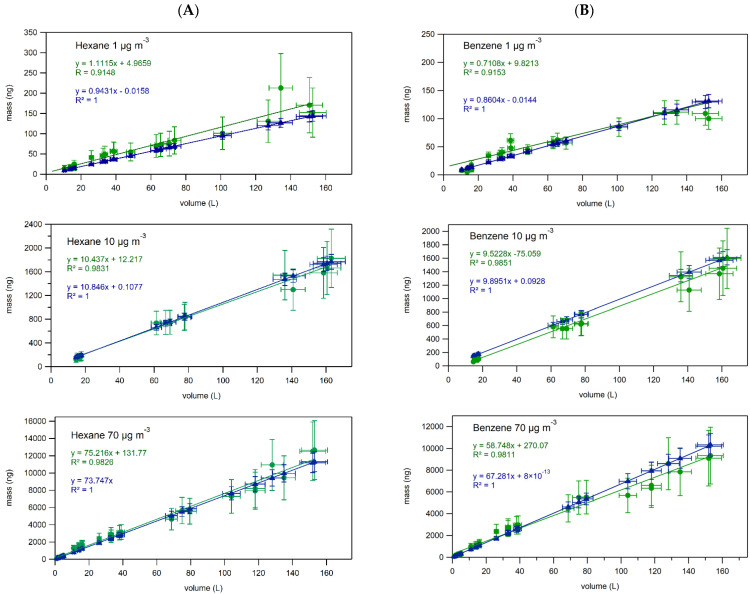
Breakthrough curves for (**A**) hexane and (**B**) benzene at concentrations of 1, 10, and 70 µg m^−3^ sampled at a flow rate ranging from 2.5 to 4.0 mL min^−1^. Green circles represent the masses measured in the TB tubes by GC-MS analysis. Blue triangles represent theoretical doped mass values.

Breakthrough studies aim to evaluate sampling efficiency by showing experimentally that 100% of the analytes of interest are adsorbed by the sampling support. Laboratory-based breakthrough studies of 10 selected NMHCs were conducted for up to 28 days at each of the 3 levels of concentrations: 1, 10, and 70 µg m^−3^. Stability of the supplied concentration was monitored by GC online. The graphs of average daily concentration versus time for hexane and benzene are depicted in Figure 1 and for other compounds of interest in Figure S1. At the level of 1 µg m^−3^, the generated concentration varied within 15% of the average value for all of the selected hydrocarbons, except for naphthalene, which showed a higher variation of ±20%. At the level of 10 µg m^−3^, the generated concentration varied within 10% of the average value, and at the level of 70 µg m^−3^ it was within 5% of the average value for all the selected hydrocarbons. The higher variation at the lowest concentration is due to the low masses of the analytes collected in the trap of the GC online (around 0.8 ng for benzene), making integration of the peaks more difficult and often performed manually. The overall stability of the generated concentrations was evaluated as satisfactory and allowed further investigations regarding breakthrough of the selected NMHCs. The results of breakthrough studies for the two lightest pollutants in this study—hexane and benzene—together with the corresponding uncertainties are represented in Figure 2 for three levels of concentration (1, 10, 70 µg m^−3^) and for other compounds of interest at minimum and maximum levels of concentration (1 and 70 µg m^−3^) in Figure S2. Practical expanded mass and volume uncertainties were applied to the results. Expanded volume uncertainty of the measured values was equal to 5%; for practical expanded mass uncertainty see Table 10. Theoretical doped mass values were calculated by multiplying the generated concentration of the analyte by the volume of gas that passed through the tube. Mass uncertainties for theoretical values ranged from 7 to 13%, taking into account the uncertainties of concentrations in the gas cylinder, uncertainty of flow generated by RDMs, and uncertainty of pumps. Theoretical volume uncertainty is 5%, as provided by the pump manufacturer.

From Figure 2 we observe excellent correspondence, within the uncertainty, of the measured values (in green) and theoretical values (in blue), suggesting the absence of breakthrough for benzene or hexane when sampling for up to 28 days under conditions of constant concentration ranging from 1 to 70 µg m^−3^ and flow rates ranging from 2.5 to 4.0 mL min^−1^. In addition, the linear relationship between measured mass in the tubes and the volume of gas that passed through the tubes further proves the absence of breakthrough phenomena (R^2^ > 0.9 for all graphs of measured mass of analyte vs. volume of gas that passed through the tube).

**Figure 3:**
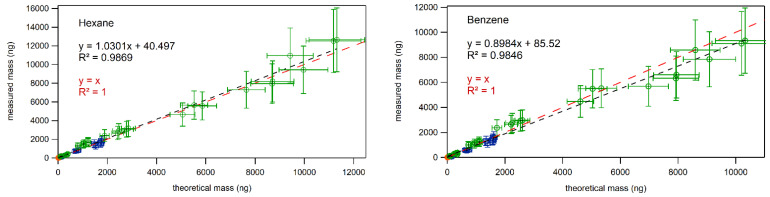
Relationship between measured and theoretical values for benzene and hexane. In orange (barely visible due to low values) are the values from breakthrough experiments performed at 1 µg m^−3^, in blue are the values from breakthrough experiments at 10 µg m^−3^, in green are the values from breakthrough experiments at 70 µg m^−3^. A dashed black line is a linear fit through the points obtained when doping the tubes with 1, 10, and 70 µg m^−3^. A dashed red line represents the 1:1 line.

Another way of analyzing the presence or absence of breakthrough is by looking at the relationship between measured values of masses of NMHCs versus theoretical values of masses applied to the sampling tubes, as can be observed in Figure 3 in the case of hexane and benzene. Absence of breakthrough is evident from (i) the linear relationship (R^2^ > 0.98) between applied and measured masses and (ii) the fact that no differences were observed between the regression line obtained from the measured mass and theoretical mass correlation and the 1:1 line.

Lighter hydrocarbons are expected to reach breakthrough earlier than heavier ones. Indeed, as expected, heavier compounds did not reach breakthrough under the defined experimental conditions. Graphs for heptane, octane, decane, toluene, ethylbenzene, m,p-xylene, o-xylene, and naphthalene are provided in Figures S2 and S3 of the Supplementary Materials. The results of the breakthrough studies suggest that the NMHCs selected for this study do not reach breakthrough limit when sampled with TB tubes and when subjected to concentrations up to 70 µg m^−3^ with a sampling time of up to 28 days and sampling volume of up to 163 L per tube. The last point of 163 L was obtained while sampling at 4.0 mL min^−1^. Taking into consideration that safe sampling time/volume represents 70% of the breakthrough time/volume, a safe sampling time of 20 days (675 h) and safe sampling volume of 114 L were chosen as a safe breakthrough limit. More studies are needed to reach the true breakthrough values on TB tubes, as they are likely to exceed the proven safety limit.

ISO 16017-1 norms recommend safe sampling volumes for hexane and benzene at 37 and 28 L, respectively, when using Chromosorb 106 (particle size 60/80 mesh, SSA = 750 m^2^ g^−1^) tubes [12]. When sampled at the lowest recommended rate of 5 mL min^−1^, these volumes correspond to safe sampling times of 123 h for hexane and 93 h for benzene. In our study, when sampling at the rate of 4 mL min^−1^ with TB tubes containing Carbograph 2, Carbograph 1, and Carboxen 1003 sorbents, the proven safe sampling volume was increased by 208% for hexane and 307% for benzene and reached 114 L. By lowering the sampling rate to 4 mL min^−1^ and validating the absence of breakthrough for up to 28 days, a 20-day-long safe sampling period for the selected NMHCs was established. The time-weighted average atmospheric concentration for a 20-day period on TB tubes is practical for evaluation of indoor air quality by cutting the number of analyses required per task. Thus, for benzene, average annual concentrations can be obtained by scheduling roughly 18 back-to-back analyses, which is relatively easy, especially when compared with 94 back-to-back 93 h samplings at 5 mL min^−1^ when a 28 L safe sampling limit is used.

In the current study, dependence of breakthrough volume on analyte concentration when sampled at the maximum flow of 4.0 mL min^−1^ for up 28 days was investigated. A classic EPA compendium method TO-17 for determination of volatile organic compounds in ambient air states there is no dependence of breakthrough volume on analyte concentration when tested on a variety of supports including tubes filled with Carbopack B and Tenax TA sorbent, at least for concentrations lower than 25 ppb [15]. Maximum concentration levels used in the current breakthrough studies expressed in ppb are those of benzene equal to 21 ppb (67 µg m^−3^) and hexane equal to 21 ppb (74 µg m^−3^). In accordance with the current TO-17 method, no dependence of breakthrough on analytes’ concentrations was observed.

Dependence of the sampling volume on RH is widely accepted. Even though in this study we did not address the influence of humidity, we evaluated the breakthrough time and volume at relevant environmental conditions of ambient temperature and 50% RH. Sampling 114 L of gas at 50% RH (at 20 °C, RH = 50% corresponds to 8.7 g m^−3^ of H_2_O content in the vapor) means that 1.0 g of water passed through the tube without triggering breakthrough. Water is known to decrease safe sampling volume and the amount adsorbed strongly depends on the type of sorbent [16]. While graphitized carbon blacks such as Carbograph 2 and Carbograph 1 are considered hydrophobic, carbon molecular sieves such as Carboxen 1003 exhibit a rather large value for maximum water sorption capacity in the range of 400–450 mg g^−1^ [17]. Graphitized carbon blacks and carbon molecular sieves both adsorb some water on the polar centers always present on the carbon surface [17]. These polar centers become saturated within seconds, and the amount of water adsorbed is much smaller than the amount necessary to form a monolayer of water and does not interfere with adsorption of analytes [17]. The high values of water sorption capacity of carbon molecular sieves are due to the presence of micropores that enable condensation of water till the pores get filled, effectively triggering earlier breakthrough [17]. Comparing the adsorbing capacity of each of the sorbents of TB tubes, it can be seen that Carbograph 2 is well suited for analysis of hydrocarbons from C8 to C20, Carbograph 1 is intended for the analysis of C5 to C12, and Carboxen 1003 is used for C-3 to C-5 compounds [18]. In the absence of compounds smaller than C-6 (hexane and benzene), the selected NMHC can be effectively retained by the first two hydrophobic sorbents and thus the breakthrough is not greatly affected by humidity. This hypothesis can be tested by repeating the protocol used in this study, but changing the humidity to higher values. It would be also interesting to test compounds smaller than C-6 to investigate whether they reach breakthrough at time periods shorter than 28 days.

As a result of the breakthrough studies, no breakthrough was observed for the 10 selected NMHCs when sampling for up to 28 days when the sampling volume did not exceed 163 L. The safe sampling volume was established at the minimum value of 114 L, which corresponds to a sampling duration of 20 days with sampling flow of 4 mL min^−1^ under laboratory conditions (RH = 50%, ambient temperature). Likewise, 100% sampling efficiency and thus uncertainty of 0% was proven experimentally for 20 days.

### 2.4. Campaign in Lyon

A 2-month-long campaign in Lyon, France tested the validated sampling method in real-life conditions where variations of pollutant concentrations due to changes in heating and ventilation of the building and the presence of additional pollutants due to vapor intrusion are expected [8,9].

About 40 hydrocarbons from 2.4-dimethylpentane to n-hexadecane were identified onsite and monitored continuously with portable GC. Major compounds included aromatics such as benzene, toluene, ethylbenzene, o-xylene, m,p-xylene, 1,2,4-trimethylbenzene, and naphthalene, and chlorinated compounds such as 1,1,1-trichloroethane. As for linear alkanes, hexane, heptane, octane, and decane were detected onsite, but in smaller concentrations than aromatics. Terpenes such as alpha pinene and limonene were also identified. The identity of these compounds was checked by analyzing the TB sampling tubes using GC equipped with an MS detector, as described in Section 3.4 (post-analysis of sampling tubes). Onsite calibration of the GC online instrument was monitored every three weeks using the cylinder containing target pollutants prepared in the lab in advance, as discussed in Section 3.1. The response coefficients of the GC online instrument were found to be stable. Most of the compounds measured by GC online were well separated, with the exceptions of n-octane that overlapped with tetrachloroethylene and isooctane that overlapped with trichloroethylene.

**Figure 4:**
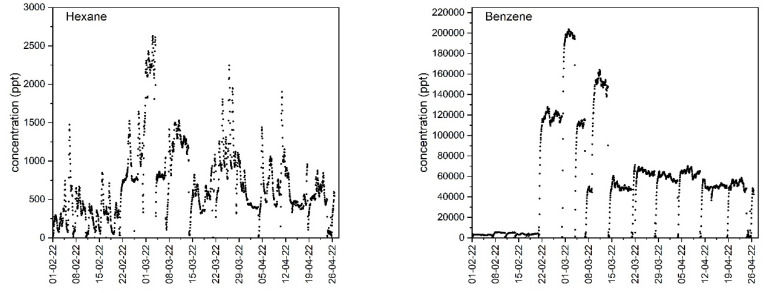
Typical profiles of hexane and benzene concentrations calculated using masses measured by portable GC analyzer. Measurements were taken continuously every hour from 1 February 2021 to 27 April 2021. Expanded uncertainty of the measurements for benzene is 25% and for hexane is 30%. Uncertainty is not applied to the graphics to facilitate observance of the trends.

An example of a temporal variation profile for benzene and hexane is provided in Figure 4. We can see that concentrations are vastly different, ranging from a maximum of 200 ppb for benzene to just 2.7 ppb for hexane. The profiles are also not the same, suggesting a difference in behavior for the two pollutants possibly due to aerobic biodegradation or difference in source depth. Note that the low concentrations, close to zero, observed on the graph correspond to the time when the door to the experimental room was open and the concentrations in the room dropped to zero. Profiles for other NMHCs are depicted in Figure S4.

In order to increase the concentrations of the pollutants to levels comfortably measured by portable GC and thus provide a solid comparison with the concentrations measured by TB tubes, openings in the floor and/or gas probes were adjusted. From 1 February to 22 February, the openings in the flooring and the gas probes were closed and the level of measured pollutant concentration was low. The influence of transfer of soil gas both through the openings in floor and one opened gas probe are visible from 22 February to 15 March, especially elevated for benzene. After 15 March, gas probes were closed and only the openings in the floor contributed to the amplification of vapor intrusion.

**Figure 5:**
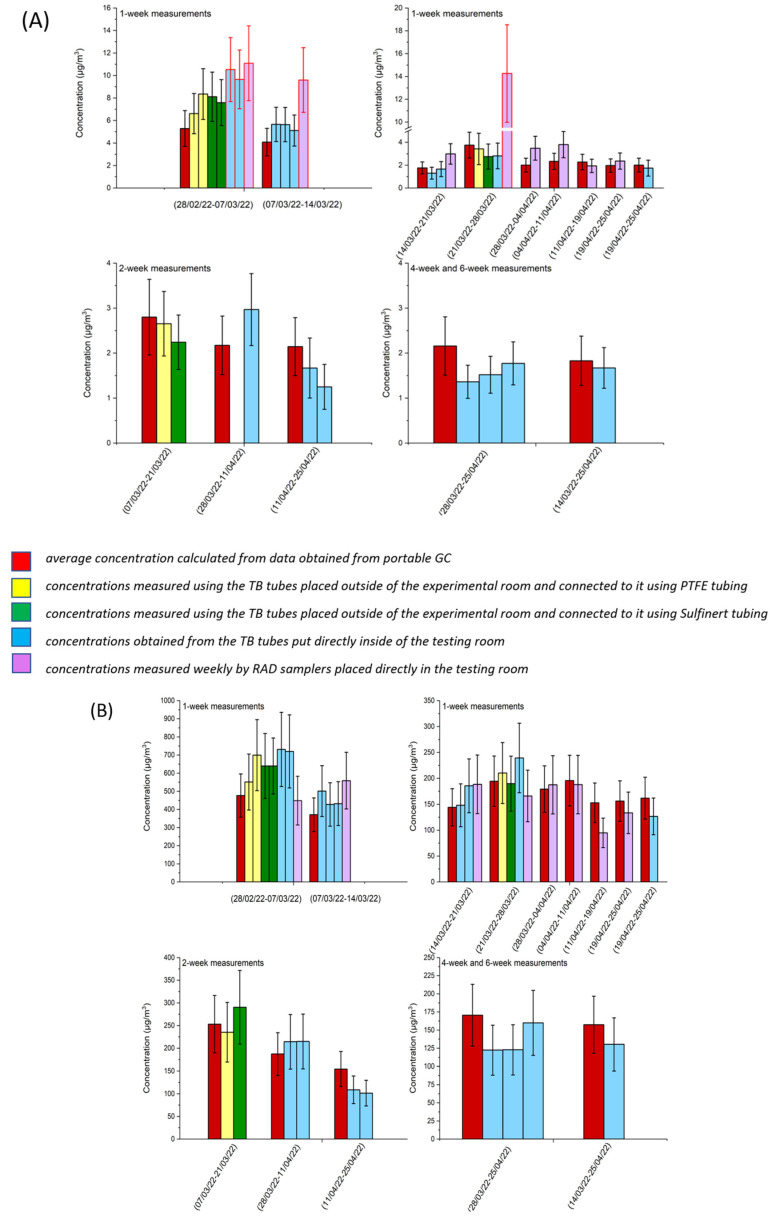
Comparison of average weekly, bi-weekly, 4-weekly, and 6-weekly concentrations for (**A**) hexane and (**B**) benzene. Red error bars represent the results that are not in accordance with the measurements obtained from portable GC.

Multiple TB tubes were placed in the experimental room for a period of 1, 2, 4, or 6 weeks (Figure 5). As explained in Section 3.5.2, the tubes were either put directly inside the testing room (blue bars) or were connected to the room with Teflon (yellow bars) or Sulfinert (green bars) tubing and placed outside the experimental room (Figure 5). Using the data from portable GC, average concentrations for the identical sampling periods were calculated (red bars). For weekly measurements, RAD passive samplers (violet bars) were put directly in the room for comparison. Figure 5 depicts the average weekly, bi-weekly, 4-weekly, and 6-weekly concentrations of hexane (A) and benzene (B). Figure S5 provides the information for the rest of NMHCs. Expanded practical concentration uncertainties were applied to the results obtained using TB tubes (see Table 13), 30% expanded uncertainty was applied to all the results obtained using RAD tubes and to all the results obtained using portable GC, except for benzene where 25% uncertainty was used for GC measurements. Bars are grouped by time periods with GC online leading the group. Measurements obtained by portable GC are considered as reference values. Data obtained from other sources are compared to data obtained from GC online.

#### 2.4.1. Comparison of Passive RAD Samplers and GC Online

In France, methods most commonly employed for indoor air measurements use RAD samplers as a preferred kind of support. In this section, concentrations measured with RAD samplers are compared with those obtained using the portable GC analyzer as a reference. The results were evaluated either as coherent (agree within the uncertainty) or incoherent (do not agree within the uncertainty). Thus, the correspondence of the results obtained using portable GC versus RAD samplers is exceptional for benzene (eight coherent results) and very good for heptane, ethylbenzene, and m,p-xylene (one case of incoherence each out of eight). The results were variable for other compounds, such as toluene (two cases of incoherence out of eight), o-xylene (two cases of incoherence out of eight), and hexane (three cases of incoherence out of eight), where RAD tended to overestimate the concentrations; or naphthalene (three cases of incoherence out of eight) and decane (four cases of incoherence out of eight), where RAD tended to underestimate the concentrations.

The incoherence may possibly be linked to the fact that, while uptake rates of some compounds, such as benzene, necessary to calculate concentration during 1-week-long passive sampling are very well studied and known to show little dependence on environmental conditions, this might not the case for other compounds. Gaps in the knowledge about the rate of passive sampling and its dependence on environmental conditions, such as humidity, can possibly lead to erroneous calculations of concentrations [19]. In addition, underestimation of the levels of VOC concentrations while using passive sampling can happen due to back diffusion [20]. Furthermore, sampling rates can be affected by higher overall concentration of VOCs, making it difficult to use passive sampling during periods of high levels of VOC emissions likely to happen during peaks of vapor intrusion [21]. Due to inherent limitations of diffusive sampling and the highly variable nature of vapor intrusion, finding alternative ways to measure the influence of vapor intrusion on air quality is highly desirable. Active sampling methods on well-adapted support present an interesting alternative as they depend only on sampled volume for as long as the sampling capacity is not reached.

#### 2.4.2. Comparison of TB Tubes and GC Online

In general, the results of the TB tubes and the values obtained by GC online are coherent and agree within the uncertainty of the measured values, no matter whether placed directly inside the experimental room or connected to the experimental room by Teflon or Sulfinert tubing. The results are excellent for benzene, toluene, ethylbenzene, and naphthalene, for which 100% coherence in the results is observed. The results are very good for heptane, hexane, and m,p-xylene, where more than 87% of the results are coherent. More specifically, out of 24 tubes only 1 tube demonstrated incoherence in the results for heptane concentrations, 2 tubes for m,p-xylene, and 3 tubes for hexane. The few observed inconsistencies were random, and underestimation and overestimation in measured concentrations of TB tubes were both noted. The biggest discrepancies in the results were demonstrated by decane and o-xylene with 9 and 7 out of 24 cases of underestimation of concentrations by TB tubes, respectively, compared with GC. The influences of (i) sampling conditions, such as humidity, temperature, and concentration of pollutants, (ii) analytical parameters, such as possible coelution with other compounds measured by GC online, and (iii) the instrumental setup itself, such as the role of tubing and variability of sampling flow, were investigated. The reasons for these inconsistencies are not clear and more investigations are necessary in order to improve long-term measurements of o-xylene and decane. Note that evaluation of the performance of TB tubes to the sampled octane was not possible due to coelution of octane with tetrachloroethylene present onsite (Section 3.1).

In addition, the fact that the concentrations measured with TB tubes and GC online for a 4-week period are coherent for the majority of compounds and especially for the lightest ones (hexane and benzene) further proves the absence of breakthrough for at least 28 days when sampling at 4 to 5 mL min^−1^. Furthermore, the results of a 6-week experiment are likewise coherent, suggesting that the breakthrough does not occur for 42 days, possibly extending the safe sampling time to 29 days, when sampling at 4.5 mL min^−1^ (safe sampling volume is equal to 70% of the breakthrough volume).

Comparing the correspondence of the results of portable GC and the results obtained by active samplers with the correspondence of the results of portable GC and the results obtained by passive samplers, active samplers are more accurate.

#### 2.4.3. Comparison of TB Tubes and Tenax TA Tubes

**Figure 6:**
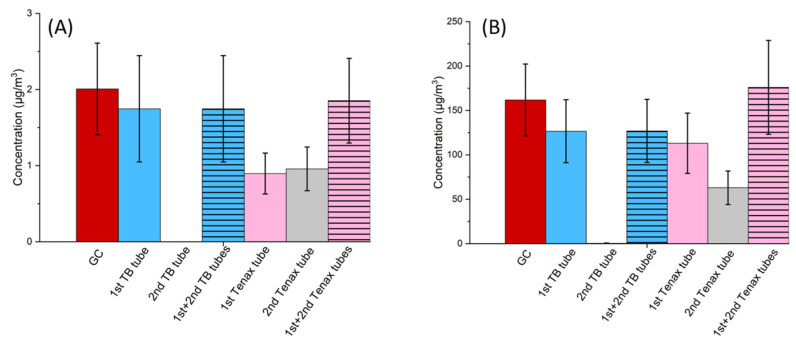
Comparison of average weekly concentrations for (**A**) hexane and (**B**) benzene when measured by GC-MS, two TB tubes connected in series, and two Tenax tubes connected in series.

In the final week of experiments, from 19 April 2022 to 25 April 2022, two TB tubes were connected in series and put directly in the experimental room to check for the absence of breakthrough phenomena. The procedure was repeated for two Tenax TA tubes for comparison (30% expanded uncertainty was applied to all the results obtained using Tenax TA tubes). From Figure 6, the breakthrough of benzene and hexane on the first Tenax TA tube can be observed, as analytes were detected in the second of the two tubes connected in series. No breakthrough was observed on the first TB tube (no analytes were detected in the second of the two tubes in series), proving the suitability of using TB tubes for measurement of lighter compounds. For compounds C7 and higher, neither Tenax TA nor TB tubes showed breakthrough. In previous studies, Gallego et al. demonstrated that Tenax TA tubes are inadequate for sampling VOCs with a boiling point of less than 100 °C, such as hexane, benzene, and heptane, due to breakthrough observed at 10 L while sampling at 70 mL min^−1^ [22]. While those observations about hexane and benzene correspond well to the results of this study, we did not observe breakthrough of heptane when sampling indoors for one week at about 5 mL min^−1^ (approximately 50 L of air). The difference in the results for heptane can be explained by the fact that a low sampling rate was employed in our study. A low sampling rate maximizes the residence time of the analyte, thus increasing the breakthrough volume.

#### 2.4.4. Results of the Campaign

The campaign in Lyon demonstrated the superior performance of TB tubes compared with RAD or Tenax tubes for measurement of selected NMHCs in real-life conditions when used for long-term continuous sampling ranging from 7 days to 6 weeks. This method is excellent for the measurement of benzene, toluene, ethylbenzene, and naphthalene (the results of GC online and TB tubes are the same within uncertainty), and very good for measurements of hexane, heptane, and m,p-xylene (more than 87% of the results are coherent). For o-xylene and decane, for which only 62% of the results are coherent, more work is necessary to understand the origin of the differences between the results provided by GC online and TB tubes and to improve their measurements. Multiple other compounds were identified during the campaign. It would be of interest to evaluate the suitability of the long-term method for testing compounds containing fewer than six and more than ten atoms of carbon, in order to monitor more pollutants in one run.

## 3. Materials and Methods

### 3.1. Gases

Certified gas cylinders were used for breakthrough investigation as a source of NMHCs. For calibration purposes, NPL primary peference material (Cylinder # D723268, National physical laboratory, Teddington Middlesex, UK) was used, consisting of a mixture of 30 components at around 200 ppb, including 8 of 10 NMHCs of interest (n-hexane, 213.3 ± 4.3 ppb; benzene, 206.2 ± 4.2 ppb; n-heptane, 213.7 ± 4.3 ppb; toluene, 200 ± 6 ppb; n-octane, 201.2 ± 4.1 ppb; ethylbenzene, 217 ± 6 ppb; meta-xylene + para-xylene, 422 ± 11 ppb; ortho-xylene, 207 ± 6 ppb). To generate the required concentrations of a mixture of hydrocarbons, a cylinder containing 10 hydrocarbons in nitrogen (n-hexane, 308 ppb; benzene, 310 ppb; n-heptane, 235 ppb; toluene, 301 ppb; n-octane, 196 ppb; ethylbenzene, 256 ppb; meta/para-xylene, 246 ppb; ortho-xylene, 248 ppb; n-decane, 197 ppb; naphthalene, 254 ppb) provided by Messer, France was used. The targeted concentration of NMHCs after dilution were 1, 10, and 70 µg m^−3^. The nominal concentrations of 8 compounds in the commercial cylinder were verified using NPL standard. They were within 10% of claimed values for all compounds. Decane and naphthalene, for which NPL standards are not available, were verified indirectly by using the theoretical response coefficient based on the response coefficient of NPL standards of octane for decane and ortho-xylene for naphthalene. While the concentration of decane was successfully verified, the concentration of naphthalene at the beginning of the experimental work is only 40% of the claimed labeled level. Experiments were performed in the following order: 1, 70, 10 µg m^−3^. The fact that the measured concentration of naphthalene in the commercial cylinder (labeled at 254 ppb) increased with time from roughly 40% to 60% of the nominal value could be due to the fact that, due to the pressure decrease inside the cylinder, naphthalene initially partially adsorbed on the walls of the cylinder was more easily released into the gas phase.

A cylinder containing a 51-component mixture (hydrocarbons and aromatics ranging from C2 to C10) at around 1 ppm each in nitrogen including NMHC of interest was purchased form Air Products, Belgium. Using this cylinder, a reference cylinder containing components at concentrations of around 40 ppb was prepared in the house and used for calibration purposes during field campaign. Concentrations of the reference cylinder were verified using NPL standard and the exact concentrations are given in Table 14. In addition, a canister containing 38 compounds likely to be found during campaign (such as benzene, toluene, ethylbenzene, m,p-xylene, o-xylene, 1,3,5-trimethymbenzene, 1,2,4-trimethylbenzene, 1,2,3-trimethylbenzene, 2-ethyltoluene, 3-ethyltoluene, 4-ethyltoluene, 1,1,1-trichloroethane, naphthalene, α-pinene, limonene, methyl cyclopentane, 3-carene, 2-methylpentane, 3-methylpentane, hexene, 2,2 dimethyl pentane, 2,4 dimethyl pentane, cyclohexane, 2-methylhexane, 2,3-dimethylpentane, nonane, decane, 2-methylbutene, hexane, heptane, octane, undecane, dodecane, tridecane, tetradecane, pentadecane, hexadecane) was prepared in the lab by liquid injection for qualitative purposes and to investigate potential overlap of the peaks. All the 38 peaks were well separated. It was observed during this work that n-octane overlapped with tetrachloroethylene and isooctane overlapped with trichloroethylene. Tetrachloroethylene and trichloroethylene were not added to the canister, but the potential overlap was noted.

### 3.2. Chemicals and Reagents

Chemicals and solvents of analytical grade were used during the research. Reference liquid solutions for gaseous doping of tubes used for studies of linearity, range, repeatability, and reproducibility came from Restek, France (Ref# 576060).

### 3.3. Sampling Tubes

For active sampling with subsequent thermal desorption and analysis by gas chromatography with mass spectrometry (GC-MS), triple-bed tubes C3-AAXX-5264 (8.9-cm × 5-mm i.d. × 6.3-mm o.d.) packed with a series of discrete beds of sorbents of increasing strength, such as Carbograph 2 (SSA = 10 m^2^ g^−1^), Carbograph 1 (SSA = 100 m^2^ g^−1^), and Carboxen 1003 (SSA = 1000 m^2^ g^−1^), were purchased from Markes Int., Bridgend, UK [23]. Triple-bed multi-sorbent tubes were chosen in order to cover a wide volatility range of HMNCs selected for the breakthrough studies (the boiling point of hexane is 69 °C, while the boiling point of naphthalene is 218 °C) and for better targeting of a wide range of analytes likely to be encountered during the field campaign [24]. Markes triple-bed tubes (TB) were used for (i) part of the analytical method development and validation studies, (ii) laboratory experiments aiming to establish the breakthrough volumes, and (iii) sampling of indoor air during the field campaign. Before and after use, the tubes were conditioned with helium at a flow rate of 50–100 mL min^−1^ under the following conditions: 1 h at 100 °C, 1 h at 200 °C, 1 h at 300 °C, and 4 h at 380 °C. Tubes were tested for the presence of 10 selected VOCs in the blanks and the results are discussed in Section 2.1.

For part of the validation studies and some campaign measurements, Tenax TA tubes (8.9-cm × 5-mm i.d. × 6.3-mm o.d) filled with 250 mg material (particle size 60/80 mesh) were prepared “in house” by TERA Environment laboratory.

In addition, Radiello 145 passive samplers (RAD145) were employed in the field campaign. RAD145 is a stainless steel net cylinder with 3 × 8 µm mesh openings and 4.8 mm diameter, packed with 350 ± 10 mg of graphitized charcoal (Carbograph 4), particle size 35–50 mesh. The RAD145 cartridge was inserted into yellow diffusive body, code RAD 1202, made of microporous polyethylene 5 mm thick with average porosity 10 ± 2 µm. The diffusive path length was 150 mm. Before use, cartridges were placed in empty inox tubes and were conditioned with nitrogen at a flow rate of 30 mL min^−1^ at 340 °C for at least 4 h using an RTA tube conditioner, TERA Environment, France. After exposure, the tubes were placed in empty glass tubes, closed with caps, and sent to TERA Environment laboratory for analysis by GC-MS.

### 3.4. Instrumentation and Chromatographic Conditions

During laboratory studies, continuous gas-phase monitoring of the generated gas flow used in the breakthrough studies was achieved using a Perkin Elmer Turbo Matrix 300 Thermal Desorber (TD) coupled with a Perkin Elmer GC CLARUS 580 gas chromatograph and FID detector. TD conditions: sampling flow at 30 mL min^−1^ for 30 min; trap adsorbent consisting of 100 mg of Carbopack B material; trap low: −30 °C; trap high: 300 °C for 15 min; no inlet split; outlet split at 7 mL min^−1^; valve at 210 °C; transfer line at 210 °C. GCFID conditions: dual columns (i) CP SIL 5CB 50 m capillary column (100% dimethylpolysiloxane (PDMS) phase) and (ii) Al_2_O _3_ Na_2_SO_4_ 50 m; carrier gas helium = 40 psi; oven 45 °C (12 min) to 200 °C (8 min) at 6 °C min^−1^; FID detector: H_2_ = 45 mL min^−1^, air = 450 mL min^−1^, T = 225 °C.

Continuous measurement of temperature (T) and relative humidity (RH) in the gas flow during the laboratory experiments was recorded using a temperature and relative humidity probe KIMO HQ 210.

Five Gillian low flow pumps were used to ensure controlled air flow between 2.5-mL min^−1^ and 4.0 mL min^−1^ through each TB tube. Three Bronkhorst flow meters were used to generate necessary concentrations of compounds of interest in 50% humid air. Gas flow was measured using the Drycal 2–500 mL and Drycal 50–5000 mL flow meters.

Atmospheric pressure readings used during the breakthrough experiments were obtained from the nearby weather station located at Avelin, 20 km from the laboratory in Douai.

During the field campaign, continuous gas-phase monitoring was achieved using a portable gas chromatographer AirmoVOC C6-C12 by Chromatotec equipped with a flame ionization detector. Expanded uncertainty of ≤25% was provided by the manufacturer for both laboratory and field measurements of benzene. A rounded value of 30% was applied to all other compounds. TD conditions: trap adsorbent 2 phase C6; trap low: +25 °C; trap high: 380 °C for 4 min; valve at 180 °C. GC conditions: column: MXT30CE 30 m, 0.25 mm, 1 μm of film ; carrier gas hydrogen ≈ 3 mL min^−1^; oven: 38 °C to 40 °C at 2 °C min^−1^, 40 °C to 50 °C at 2 °C min^−1^, 50 °C to 80 °C at 10 °C min^−1^, 80 °C to 220 °C at 15 °C min^−1^, 220 °C to 230 °C at 2 °C min^−1^, 230 °C to 269 °C at 9 °C min^−1^. FID detector: H2 = 27 mL min^−1^, air = 180 mL min^−1^, T = 200 °C. The portable GC provided 24 chromatograms per day for the duration of 2 months.

Post-analysis of sampling tubes was performed offsite by TERA Environment laboratory using Perkin Elmer TD-GC/MS (thermal desorber coupled with Perkin Elmer CLARUS 680 gas chromatograph and Perkin Elmer Clarus SQ8T mass spectrometer detector). TD conditions: trap adsorbent 30 mg Carbopack B and 100 mg Carbosieve; tube purge: 1min; tube desorb: 40 mL min^−1^ at 350 °C for 20 min; trap low: +5 °C; trap high: 300 °C for 10 min; inlet split 5 mL min^−1^ or 50 mL min^−1^; outlet split at 5 mL min^−1^ or 50 mL mL min^−1^; valve at 225 °C; transfer line at 275 °C. GC conditions: column: DB 5MS 60 m 0.25mm 1 μm de phase (5% phenyl—méthylpolysiloxane); carrier gas helium = 15 psi; oven 40 °C to 300°C at 10 °C min^−1^. MS conditions: mass range: 35 to 300 Da, source temperature: 300 °C, transfer line at 200 °C; photomultiplier at around 2000 Volts.

### 3.5. Experimental Procedure

#### 3.5.1. Laboratory Studies

The analytical method for determination of NMHCs using active sampling TB tubes (sorbent: Carbograph 2TD/Carbograph 1 TD/Carboxen 1003) was developed and validated by TERA Environment laboratory. The analytical method is described above. Note that long-term sampling can result in accumulation of a large amount of water in the sampling tubes. The accumulated water can generate ice plugs during cryogenic focusing of thermally desorbed compounds or blow out an FID flame [21]. By purging the tube and increasing the temperature of the trap to above 0 °C, accumulation of ice on the trap was successfully prevented. The analytical part of the method was validated for linearity, range, and repeatability using Tenax tubes. Empty stainless steel tubes were used for determination of the LOQ/LOD of the analytical system. Sampling support was evaluated for accuracy and stability using TB tubes (Section 2.1.2). Overall validation data were used to calculate the overall uncertainty of the method (Section 2.2).

Breakthrough experiments were designed to prove the sampling efficiency of TB tubes by evaluating the volume of a flowing gas containing ten chosen hydrocarbons that passed though the analytical support under a defined set of conditions till each hydrocarbon of interest saturated the analytical support and started eluting from it. This volume depends on the species being sampled along with environmental parameters, such as humidity and temperature [25]. Lighter NMHCs are expected to have smaller breakthrough volumes. High levels of humidity and elevated temperatures might also negatively affect breakthrough volumes. Seventy percent of the breakthrough volume is a value accepted as a safe sampling volume [26].

**Figure 7:**
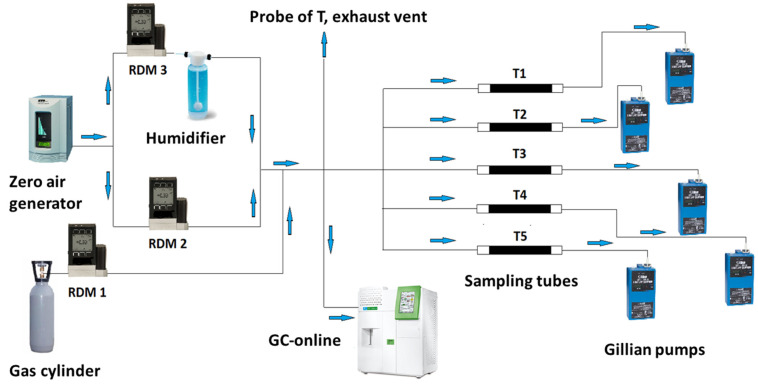
Experimental laboratory setup to evaluate breakthrough volume of ten selected NMHCs.

Throughout the laboratory experimental work, generated levels of concentration (1, 10, and 70 µg m^−3^) of ten selected NMHCs were monitored using an online gas chromatography system calibrated for each level of concentration by means of the NPL reference standard. All the experiments were run under conditions of 50% RH and ambient temperature and pressure. The detailed setup and experimental conditions are shown in Figure 7 and Table 15. Swagelok Inox 316 connectors and Restek Sulfinert tubing of 6.35 mm o.d./5.33 mm i.d. and 3.18 mm o.d./1.40 mm i.d. were used for the setup. Teflon FEP (fluorinated ethylene propylene) tubing of 6 mm o.d. and 4 mm i.d. was used for the supply of humid air. During each experiment, five active TB sampling tubes were connected to the generated gas supply in parallel and five designated Gillian low flow pumps ensured controlled air flow through each tube. Pumps were regulated to ensure flow between 2.5- and 4.0-mL min^−1^. The flow rate through each pump was measured before and after each experiment and the average flow rate for each pump was then calculated. The curves of mass of the analyte measured in the tube vs. volume of air that passed through the tube were constructed for each compound. Breakthrough is expected when the relationship between the two values starts deviating from the linear.

#### 3.5.2. Field Investigations

**Figure 8:**
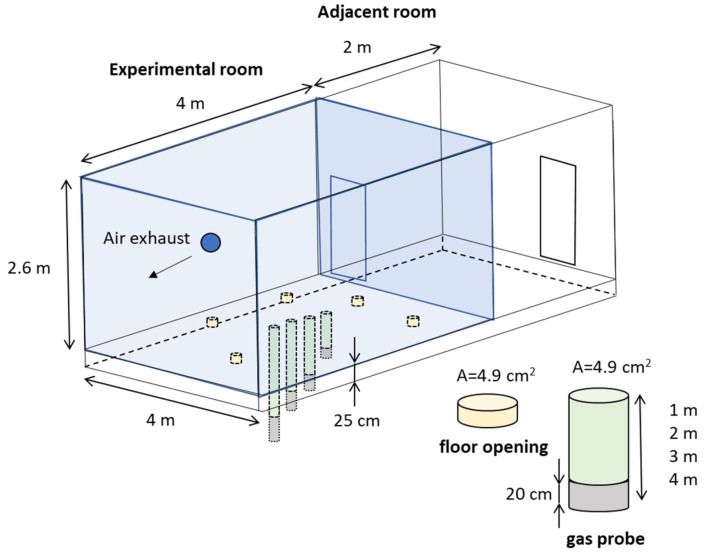
Experimental setup for the Lyon campaign to measure indoor concentrations of NMHCs. In blue is the experimental room with dimensions of 4 × 4 × 2.6 m (L × W × H); flooring is 25 cm thick. Adjacent room measures 4 × 2 m (L × W) and has no ceiling. Five yellow cylinders represent openings with area of 4.9 cm^2^ and 25 cm deep made in the floor of the experimental room to increase permeability. Four green cylinders represent gas probes with 20 cm bottom openings depicted in grey, which are installed to sample soil gas at different depths: 1, 2, 3, and 4 m below the slab.

A field campaign in the city of Lyon was organized to test the performance of TB tubes in real-life conditions. Between the years 1870 and 1920, the site chosen for the campaign was used as an asphalt and tar manufacturing facility. The site was then converted into a washing machine production site where activity continued till 2011. To study the transfer of pollutants from soil into the indoor air, two adjacent rooms were constructed inside a former industrial hall (Figure 8). The first room measured 4 by 4 m and was 2.6 m high. It was used as the experimental room where sampling of indoor air took place. The second adjacent room was 2 by 4 m and had no ceiling, thus opening directly into the industrial hall. The second adjacent room was where the instrumentation was placed, including the online portable GC, the hydrogen generator, some of the pumps, etc. The walls and the ceiling of the experimental room were covered from the inside with thin aluminum sheets to limit adsorption of VOCs on these surfaces. Five floor openings of 4.9 cm^2^ area and 25 cm depth (corresponding to floor thickness of 25 cm) were made in the floor of the experimental room to increase permeability and to facilitate the transfer of the pollutants. In addition, four gas probes made of high-density polyethylene with 20 cm bottom openings were installed to sample soil gas at different depths: 1, 2, 3, and 4 m below the slab (sampling of soil gas is not discussed in this article). The experimental room was equipped with a mechanical air exhaust to create adapted air exchange and depressurization within the room facilitating vapor intrusion from soil. The extraction rate was equal to 10 ± 1 m^3^ h^−1^ and the indoor–outdoor depressurization was equal to 20 ± 2 Pa.

On 1 February, a portable GC by Chromatotec was installed onsite in the adjacent room where the concentrations of various VOCs in the indoor air were continuously monitored. The campaign started at the end of February and lasted till the end of April. The values from the portable GC are used as a reference. Sulfinert tubing of roughly 4 m length and 3.18 mm o.d./1.40 mm i.d. led from the center of the experimental room to the portable GC. The portable GC instrument sampled the air at 65 mL min^−1^ and an additional pump was installed upstream to increase sampling of air through the tubing to 200 mL min^−1^, to limit the residence time of analytes in the tubing to less than 0.1 min.

In addition, RAD145 passive samplers, a commonly used kind of support for characterization of vapor intrusion in commercial studies, specifically conceived for long-term sampling of BTEX and other VOCs in urban environments with subsequent thermal desorption were placed for one week in the experimental room. Sampling rates for Radiallo145 were obtained experimentally and are listed in the Radiello instruction manual or, in case of naphthalene, are based on theoretical calculations by the Maugeri foundation (Table S1 of Supplementary Materials Section) [21]. These rates were corrected for the temperature by using the following equation:

$$Q_{K}=Q_{298}{\left(\frac{K}{298}\right)}^{1.5}$$

where $Q_{K}$ is the sampling rate at the temperature *K* and $Q_{298}$ is the reference value at 298 K (25 °C). As a result of temperature variations, for every ±10 °C from the reference value, variation in sampling rates is expected to change by ±5%.

Initially, all sampling TB tubes onsite were supposed to be placed in the adjacent room and connected to the sampling room via tubing to allow sampling monitoring without disturbing pollutant transfer in the room. However, based on preliminary results, the setup was modified to include tubes placed directly in the experimental room in order to limit the influence of tubing. Thus, TB tubes were employed for various time periods, placed directly in the experimental room or in the adjacent room (Figure 8, Table 16) with sampling using different kinds of tubing leading to the experimental room. More specifically, TB tubes were placed in the adjacent room and connected to the sampling room by two types of tubing: (i) relatively inert, commonly used for soil gas sampling, Teflon FEP tubing (6 mm o.d., 4 mm i.d., 3 m length) and (ii) inert Sulfinert silicon-coated tubing (3.18 mm o.d./1.40 mm i.d., 3 m length). Additional information on exact periods of sampling, type of sampling support (TB, Tenax, and Radiallo), tubing material (Teflon, Sulfinert, or none), average sampling flows (from 4 to 5 mL min^−1^), total sampled volumes (from 40 to 274 L), residence time of analytes in the tubing, temperature, and humidity of experimental adjacent rooms can be found in Table 16.

At the end of the campaign, two TB tubes were put in series to test for the presence of compounds of interest in the second tube, which would suggest a breakthrough. At the same time, two Tenax tubes were also put in series to compare the capacity of the two supports.

## 4. Conclusions

A reliable long-term low-flow (at around 4 mL min^−1^) sampling method for determination of NMHCs in indoor air was developed for 10 NMHCs: hexane, benzene, heptane, toluene, octane, ethylbenzene, m,p-xylene, o-xylene, decane, and naphthalene. The method was successfully validated for linearity and range, repeatability, LOQ/LOD, reproducibility, accuracy, and stability. At 50% RH, breakthrough did not occur in the time frame of 28 days/163 L of sampling at 4 mL min^−1^ for any of the tested compounds. Thus, in accordance with ISO 16017-1, 20 days/114 L was established as safe sampling time/volume when sampling at 4 mL min^−1^ [12]. The safe sampling volume is limited by the breakthrough of the lightest compounds (benzene and hexane) and is longer for the heavier compounds. These results point to a considerable improvement in the measurement of annual concentrations of benzene, as long-term 20-day sampling at 4 mL min^−1^ reduces by 5 times the amount of analysis necessary to obtain the data (from 94 to 18 back-to-back samplings). The safe sampling time/volume of 20 days/114 L is likely to be higher, as excellent correspondence in the results for the TB tubes compared with the reference values was demonstrated during the field campaign in a 42-day experiment when sampling at 4.5 mL min^−1^, corresponding to the safe sampling volume of 29 days.

Using the developed method, a field campaign was organized at a former industrial site in Lyon with proven underground hydrocarbon pollution. The deployment of TB tubes for long-term sampling was found to be easy and required minimum manpower. Calculations of concentrations in TB tubes are straightforward and depend only on time and sampled volume. In general, within uncertainty, a good correspondence of the concentrations measured via TB sampling tubes and portable GC was established. A preference for active sampling using TB tubes in comparison with passive radial sampling was demonstrated for a number of compounds, especially for naphthalene.

## Figures and Tables

**Table 1:** LOQ and LOD of the analytical method for determination of 10 selected NMHCs derived by using empty tubes.

	Limit of Detection (ng)	Limit of Quantification (ng)
hexane	0.11	0.40
benzene	0.22	1.00
heptane	0.16	0.57
toluene	0.44	1.60
octane	0.08	0.31
ethylbenzene	0.08	0.30
meta + para-xylene	0.02	0.04
o-xylene	0.03	0.11
decane	0.06	0.24
naphthalene	0.01	0.04

**Table 2:** Linearity studies using Tenax tubes. Determination coefficients (R^2^) of 6 plots of peak area vs. mass for 10 analytes at low and high levels of mass on the tube.

	**Low Level (5 ng to 200 ng)**						**High Level (500 ng to 10,000 ng)**					
	**Plot 1**	**Plot 2**	**Plot 3**	**Plot 4**	**Plot 5 **	**Plot 6**	**Plot 1**	**Plot 2**	**Plot 3**	**Plot 4**	**Plot 5**	**Plot 6**
hexane	0.9908	0.9944	0.9953	0.9988	0.9974	0.9996	0.9901	0.9952	0.9991	0.9972	0.9975	
benzene	0.9974	0.9902	0.9971	0.9964	0.9996	0.9964	0.9898	0.9958	0.9929	0.9988	0.9967	0.9936
heptane	0.9915	0.9950	0.9992	0.9994	0.9943	0.9978	0.9963	0.9999	0.9976	0.9998	0.9997	
toluene	0.9943	0.9929	0.9912	0.9969	0.9977	0.9983	0.9997	0.9973	0.9892	0.9892	0.9991	0.9908
octane	0.9958	0.9957	0.9997	0.9921	0.9979	0.9977	0.9989	0.9998	0.9988	0.9996	0.9986	0.9986
ethylbenzene	0.9993	0.9919	0.9974	0.9879	0.9971	0.9981	0.9894	0.9886	0.9998	0.9962	0.9998	0.9906
o-xylene	0.9952	0.9894	0.9992	0.992	0.9988	0.9985	0.9995	0.9985	0.9980	0.9987	0.9972	
decane	0.9892	0.9977	0.9999	0.9981	0.9979	0.9964	0.9975	0.9969	0.9996	0.9972		
naphthalene	0.9979	0.9963	0.9985	0.9991	0.9988	0.9998	0.9962	0.9896	0.9989	0.9930	0.9952	
	**Low level (10 ng to 400 ng)**						**High level (1000 ng to 20,000 ng)**					
	**Plot 1**	**Plot 2**	**Plot 3**	**Plot 4**	**Plot 5**	**Plot 6**	**Plot 1**	**Plot 2**	**Plot 3**	**Plot 4**	**Plot 5**	**Plot 6**
meta + para-xylene	0.9988	0.9976	0.9913	0.9986	0.9957	0.9986	0.9995	0.9985	0.998	0.9987	0.9972	

**Table 3:** Repeatability of the analytical method for determination of 10 selected NMHCs derived by using Tenax tubes and expressed as %RSD of 6 measured mass values.

	Low Level					High Level				
	5 ng	20 ng	40 ng	100 ng	200 ng	500 ng	1000 ng	2000 ng	5000 ng	10,000 ng
hexane	6.1%	6.7%	3.9%	7.6%	7.4%	8.4%	9.3%	8.1%	8.2%	9.9%
benzene	12.2%	5.7%	6.5%	8.1%	7.0%	9.7%	8.1%	8.9%	4.6%	11.2%
heptane	7.4%	6.9%	3.5%	8.1%	9.6%	9.2%	8.0%	8.8%	7.3%	7.9%
toluene	8.8%	9.5%	6.5%	7.2%	7.6%	9.6%	7.1%	9.6%	6.1%	9.8%
octane	3.2%	8.4%	4.5%	9.8%	9.5%	5.4%	8.8%	6.2%	9.4%	8.6%
ethylbenzene	5.5%	9.7%	6.4%	8.3%	6.8%	7.9%	9.6%	9.1%	7.3%	8.4%
o-xylene	9.7%	7.6%	5.9%	8.5%	8.0%	8.7%	8.1%	10.0%	6.0%	6.1%
decane	13.7%	7.3%	9.6%	8.7%	6.4%	9.4%	4.8%	9.9%	9.0%	4.0%
naphthalene	8.5%	14.0%	8.6%%	7.0%	7.0%	8.0%	8.7%	7.5%	3.9%	7.1%
	**10 ng**	**40 ng**	**80 ng**	**200 ng**	**400 ng**	**1000 ng**	**2000 ng**	**4000 ng**	**10,000 ng**	**20,000 ng**
meta + para-xylene	7.1%	3.5%	5.2%	9.6%	7.0%	7.6%	8.3%	8.5%	6.9%	8.4%

**Table 4:** Masses of 10 selected NMHCs in the blanks of the unexposed TB tubes.

Compound	Mass Average of 6 Tubes (ng)	Compound	Mass Average of 6 Tubes (ng)
hexane	<LOD	benzene	1.0
heptane	<LOD	toluene	<LOD
octane	<LOD	ethylbenzene	<LOD
meta + para-xylene	<LOD	o-xylene	<LOD
decane	<LOD	naphthalene	<LOD

**Table 5:** Accuracy of the entire method for determination of 10 selected NMHCs evaluated as %recovery using TB tubes.

	**Low Level**					**High Level**				
	**5 ng**	**20 ng**	**40 ng**	**100 ng**	**200 ng**	**500 ng**	**1000 ng**	**2000 ng**	**5000 ng**	**10,000 ng**
hexane	134.2%	118.2%	127.8%	124.2%	128.5%	104.8%	102.0%	104.9%	122.9%	118.8%
benzene	128.4%	105.8%	105.5%	102.4%	97.0%	117.6%	121.1%	117.5%	108.4%	113.9%
heptane	93.7%	102.7%	88.2%	101.9%	110.0%	75.8%	74.4%	76.8%	104.3%	126.6%
toluene	97.3%	86.7%	84.9%	85.5%	90.4%	103.6%	102.4%	107.2%	109.9%	109.9%
octane	105.6%	91.7%	91.5%	93.2%	101.2%	89.4%	81.8%	87.6%	91.6%	116.8%
ethylbenzene	86.8%	78.9%	75.1%	78.14%	85.1%	95.3%	97.6%	100.3%	107.2%	111.1%
o-xylene	83.6%	76.2%	71.0%	72.4%	79.9%	94.7%	95.1%	95.9%	110.1%	100.3%
decane	110.3%	91.5%	93.1%	91.1%	102.0%	77.7%	79.9%	85.0%	92.1%	111.9%
naphthalene	136.0%	96.1%	92.5%	89.8%	84.0%	119.6%	104.8%	101.0%	93.8%	106.5%
	**Low level**					**High level**				
	**10**	**40**	**80**	**200**	**400**	**1000**	**2000**	**4000**	**10,000**	**20,000**
meta + para-xylene	89.9%	84.4%	78.7%	81.5%	87.1%	95.3%	97.2%	102.0%	109.8%	111.9%

**Table 6:** Reproducibility of the entire method for determination of 10 selected NMHCs derived by doping TB tubes and expressed as %RSD of 6 measured mass values.

	**Low Level**					**High Level**				
	**5 ng**	**20 ng**	**40 ng**	**100 ng**	**200 ng**	**500 ng**	**1000 ng**	**2000 ng**	**5000 ng**	**10,000 ng**
hexane	20.4%	18.0%	13.1%	8.1%	7.3%	17.1%	10.9%	14.9%	11.6%	18.1
benzene	10.4%	8.9%	8.2%	6.4%	7.0%	6.5%	11.0%	10.1%	5.0%	19.0%
heptane	24.2%	19.5%	17.0%	18.4%	17.9%	11.6%	18.2%	17.7%	6.9%	16.3%
toluene	12.1%	9.7%	15.3%	8.3%	8.6%	5.7%	6.9%	8.0%	11.1%	15.9%
octane	9.5%	6.7%	10.7%	7.6%	6.3%	22.6%	5.2%	8.1%	18.8%	19.9%
ethylbenzene	11.6%	6.6%	19.2%	7.3%	6.4%	9.5%	14.8%	14.1%	14.5%	11.1%
o-xylene	10.8%	6.0%	19.9%	10.2%	9.7%	12.3%	12.8%	15.3%	11.9%	8.4%
decane	16.4%	15.3%	14.3%	9.8%	5.5%	13.4%	11.1%	13.4%	17.0%	17.1%
naphthalene	21.3%	14.3%	17.0%	11.0%	5.6%	9.4%	16.4%	17.9%	19.5%	16.9%
	**Low level**					**High level**				
	**10 ng**	**40 ng**	**80 ng**	**200 ng**	**400 ng**	**1000 ng**	**2000 ng**	**4000 ng**	**10,000 ng**	**20,000 ng**
meta + para-xylene	10.6%	6.5%	19.7%	9.8%	7.7%	7.6%	9.4%	9.9%	13.6%	15.2%

**Table 7:** Stability of the entire method for determination of 10 selected NMHCs derived by doping TB tubes, expressed as %ratio of average mass of freshly doped tubes over tubes subjected to 21-day storage.

	**Low Level; 200 ng**	**High Level; 10,000 ng**
hexane	82.1%	98.6%
benzene	93.3%	97.4%
heptane	90.3%	84.7%
toluene	104.6%	91.7%
ethylbenzene	111.1%	82.8%
o-xylene	112.1%	105.4%
decane	92.9%	115.2%
naphthalene	117.4%	86.6%
	**Low level; 400 ng**	**High level; 20,000 ng**
meta + para-xylene	105.5%	90.5%

**Table 8:** Bias of analyte recoveries ($B_{a})$ based on six measurements per each mass level. Data obtained from accuracy studies using TB tubes.

	**Low Level**					**High Level**				
	**5 ng**	**20 ng**	**40 ng**	**100 ng**	**200 ng**	**500 ng**	**1000 ng**	**2000 ng**	**5000 ng**	**10,000 ng**
hexane	0.34	0.18	0.28	0.24	0.29	0.05	0.02	0.05	0.23	0.19
benzene	0.28	0.06	0.05	0.02	0.03	0.18	0.21	0.18	0.08	0.14
heptane	0.06	0.03	0.12	0.02	0.10	0.24	0.26	0.23	0.04	0.27
toluene	0.03	0.13	0.15	0.15	0.10	0.04	0.02	0.07	0.10	0.10
octane	0.06	0.08	0.09	0.07	0.01	0.11	0.18	0.12	0.08	0.17
ethylbenzene	0.13	0.21	0.25	0.22	0.15	0.05	0.02	0.00	0.07	0.11
o-xylene	0.16	0.24	0.29	0.28	0.20	0.05	0.05	0.04	0.10	0.00
decane	0.10	0.09	0.07	0.09	0.02	0.22	0.20	0.15	0.08	0.12
naphthalene	0.36	0.04	0.08	0.10	0.16	0.20	0.05	0.01	0.06	0.06
	**Low level**					**High level**				
	**10 ng**	**40 ng**	**80 ng**	**200 ng**	**400 ng**	**1000 ng**	**2000 ng**	**4000 ng**	**10,000 ng**	**20,000 ng**
meta + para-xylene	0.10	0.16	0.21	0.19	0.13	0.05	0.03	0.02	0.10	0.12

**Table 9:** Standard mass uncertainties of 10 NMHCs at 10 levels of mass determined in the TB tube.

	**Low Level**					**High Level**				
	**5 ng**	**20 ng**	**40 ng**	**100 ng**	**200 ng**	**500 ng**	**1000 ng**	**2000 ng**	**5000 ng**	**10,000 ng**
hexane	0.25	0.18	0.20	0.19	0.21	0.12	0.11	0.11	0.17	0.17
benzene	0.21	0.09	0.09	0.10	0.09	0.15	0.16	0.14	0.08	0.16
heptane	0.14	0.12	0.12	0.13	0.15	0.20	0.21	0.20	0.12	0.21
toluene	0.11	0.13	0.13	0.12	0.11	0.12	0.10	0.12	0.11	0.14
octane	0.07	0.11	0.09	0.12	0.11	0.14	0.15	0.12	0.15	0.17
ethylbenzene	0.13	0.17	0.19	0.17	0.13	0.14	0.15	0.15	0.15	0.15
o-xylene	0.16	0.18	0.21	0.20	0.16	0.11	0.11	0.13	0.11	0.08
decane	0.17	0.12	0.13	0.12	0.09	0.19	0.16	0.17	0.15	0.14
naphthalene	0.26	0.19	0.16	0.15	0.16	0.17	0.14	0.13	0.13	0.13
	**Low level**					**High level**				
	**10 ng**	**40 ng**	**80 ng**	**200 ng**	**400 ng**	**1000 ng**	**2000 ng**	**4000 ng**	**10,000 ng**	**20,000 ng**
meta + para-xylene	0.11	0.14	0.17	0.16	0.13	0.11	0.11	0.11	0.12	0.14

**Table 10:** Standard and expanded practical mass uncertainties of 10 NMHCs for 3 ranges of mass determined in the tube.

	**<20 ng**		**20–200 ng**		**201–10,000 ng**	
	**Standard**	**Expanded**	**Standard**	**Expanded**	**Standard**	**Expanded**
hexane	0.25	0.49	0.20	0.40	0.13	0.27
benzene	0.21	0.43	0.09	0.19	0.14	0.28
heptane	0.14	0.29	0.13	0.26	0.19	0.37
toluene	0.11	0.22	0.12	0.25	0.12	0.24
octane	0.07	0.15	0.11	0.21	0.15	0.29
ethylbenzene	0.13	0.25	0.17	0.33	0.15	0.30
o-xylene	0.16	0.32	0.19	0.38	0.11	0.22
decane	0.17	0.34	0.11	0.23	0.16	0.33
naphthalene	0.26	0.53	0.16	0.32	0.14	0.28
	**<20 ng**		**20–400 ng**		**401–20,000 ng**	
	**standard**	**expanded**	**standard**	**expanded**	**standard**	**expanded**
meta + para-xylene	0.11	0.21	0.15	0.30	0.12	0.24

**Table 11:** Individual volume uncertainties for 80 laboratory experiments lasting from 8 h to 28 days, intended to explore breakthrough volume.

Run	8 h	1 d		3 d			7 d		14 d			21 d		28 d	
	80 µg/m^3^	1 µg/m^3^	80 µg/m^3^	1 µg/m^3^	10 µg/m^3^	80 µg/m^3^	1 µg/m^3^	80 µg/m^3^	1 µg/m^3^	10 µg/m^3^	80 µg/m^3^	80 µg/m^3^	1 µg/m^3^	10 µg/m^3^	80 µg/m^3^
1	0.0027	0.0060	0.0040	0.0248	0.007	0.0100	0.0085	0.0079	0.0106	0.0105	0.0102	0.0269	0.0139	0.0103	0.017
2	0.0028	0.0074	0.0041	0.0169	0.0154	0.0089	0.0101	0.0079	0.0095	0.0105	0.01	0.0106	0.0114	0.0114	0.0219
3	0.0044	0.0076	0.0067	0.0120	0.0281	0.0053	0.0079	0.0090	0.0103	0.0141	0.0099	0.0101	0.0131	0.0095	0.0151
4	0.0039	0.0056	0.0055	0.0149	0.0066	0.0088	0.0085	0.0079	0.0146	0.0089	0.0108	0.0101	0.0120	0.0089	0.0133
5	0.0030	0.0070	0.0045	0.0127	0.0066	0.0084	0.0104	0.0091	0.0128	0.0089	0.0106	failed	0.0135	0.0108	failed
6											0.0048				
7											0.0177				
8											0.0691				
9											0.005				
10											failed				
#measurements	5	10		15			10		15			5	14		
Average	0.0034	0.0058		0.012			0.009		0.011			0.014	0.013		
Maximum	0.0044	0.0076		0.0281			0.010		0.069			0.027	0.022		
Minimum	0.0027	0.004		0.0053			0.008		0.005			0.0101	0.009		
SD	0.0008	0.0013		0.0067			0.0009		0.0017			0.0083	0.0034		
RSD	22.33%	22.85%		53.97%			10.63%		15.74%			57.68%	25.95%		

**Table 12:** Standard concentration uncertainties of 10 NMHCs at 10 levels of mass determined in the tube.

	**Low Level**					**High Level**				
	**5 ng**	**20 ng**	**40 ng**	**100 ng**	**200 ng**	**500 ng**	**1000 ng**	**2000 ng**	**5000 ng**	**10,000 ng**
hexane	0.25	0.18	0.21	0.20	0.21	0.12	0.11	0.11	0.17	0.17
benzene	0.22	0.09	0.10	0.10	0.10	0.15	0.16	0.15	0.08	0.16
heptane	0.15	0.13	0.12	0.13	0.15	0.20	0.21	0.20	0.13	0.21
toluene	0.11	0.14	0.13	0.12	0.11	0.12	0.10	0.13	0.11	0.14
octane	0.08	0.11	0.09	0.12	0.11	0.14	0.16	0.13	0.15	0.17
ethylbenzene	0.13	0.17	0.19	0.17	0.14	0.14	0.16	0.15	0.15	0.16
o-xylene	0.16	0.18	0.21	0.20	0.17	0.12	0.11	0.13	0.11	0.09
decane	0.17	0.12	0.13	0.12	0.09	0.19	0.16	0.17	0.16	0.14
naphthalene	0.26	0.19	0.16	0.15	0.16	0.17	0.14	0.14	0.13	0.14
	**Low level**					**High level**				
	10	40	80	200	400	1000	2000	4000	10,000	20,000
meta + para-xylene	0.11	0.14	0.17	0.16	0.13	0.11	0.11	0.12	0.12	0.14

**Table 13:** Standard and expanded practical concentration uncertainties of 10 NMHCs for 3 ranges of mass determined in the tube.

	**<20 ng**		**20–200 ng**		**201–10,000 ng**	
	**Standard**	**Expanded**	**Standard**	**Expanded**	**Standard**	**Expanded**
hexane	0.25	0.50	0.20	0.40	0.14	0.27
benzene	0.22	0.43	0.10	0.19	0.14	0.28
heptane	0.15	0.29	0.13	0.27	0.19	0.38
toluene	0.11	0.22	0.13	0.25	0.12	0.24
octane	0.08	0.15	0.11	0.22	0.15	0.30
ethylbenzene	0.13	0.26	0.17	0.34	0.15	0.30
o-xylene	0.16	0.33	0.19	0.38	0.11	0.22
decane	0.17	0.35	0.12	0.23	0.17	0.33
naphthalene	0.26	0.53	0.16	0.33	0.14	0.29
	**<20 ng**		**20–400 ng**		**401–20,000 ng**	
	**standard**	**expanded**	**standard**	**expanded**	**standard**	**expanded**
meta + para-xylene	0.11	0.22	0.15	0.30	0.12	0.24

**Table 14:** Concentrations of selected NMHCs in a gas cylinder prepared in IMT laboratory and used for calibration during field campaign.

Compound	Concentration (ppb)	Compound	Concentration (ppb)	Compound	Concentration (ppb)
3-methylpentane	36.39	cyclohexane	23.98	o-xylene	10.83
hexene	38.42	2-methylhexane	77.17	n-nonane	14.07
n-hexane	39.27	iso-octane	29.68	1,3,5-trimethylbenzene	7.89
2,2-dimethylpentane	37.90	n-heptane	35.22	1,2,4-trimethylbenzene	6.91
2,4-dimethylpentane	39.31	toluene	21.59	n-decane	9.43
2,2,3-trimethylbutane	41.62	n-octane	22.44	1,2,3-trimethylbenzene	6.38
benzene	34.45	ethylbenzene	14.51		
3,3-dimethylpentane	39.63	m,p-xylenes	24.58		

**Table 15:** Experimental conditions for studies investigating breakthrough volume of ten NMHCs.

Target Concentration Level	Low (1 µg m^−3^)	Medium (10 µg m^−3^)	High (70 µg m^−3^)
Individual Concentrations (µg m^−3^)			
n-hexane	0.9	10.9	73.7
benzene	0.9	9.9	67.3
n-heptane	0.8	9.6	65.4
toluene	1.0	11.3	77.1
n-octane	0.8	9.2	62.2
ethylbenzene	1.0	11.1	75.5
meta or para-xylene	0.9	10.7	72.6
ortho-xylenes	0.9	10.8	73.2
n-decane	1.0	11.5	77.9
naphthalene	0.5	7.5	38.8
# of time periods investigated	5	3	7
time periods of individual runs (hr)	24, 72, 168, 336, 668	72, 334, 672	8, 23, 72, 168, 330, 499, 672
time periods of individual runs	24 h, 3 d, 7 d, 14 d, 28 d	3 d, 14 d, 28 d	8 h, 1 d, 3 d, 7 d, 14 d, 21 d, 28 d
number of samplers for each run	5	5	5
T (°C)	ambient	ambient	ambient
RH	50	50	50

**Table 16:** Tube employment and conditions during campaign to measure indoor concentration of NMHCs in Lyon.

Period	In/out of Experimental Room Experiment	Sampling Support	Tubing Material	Sampled Volume (L)	Average Flow (mL/min)	Residence Time of Analytes in the Tub (min)	Experimental Room T (°C)	Experimental Room RH (%)	Adjacent Room T (°C)	Adjacent Room RH (%)	Special Comments
One week											
28 February 2022–7 March 2022	out	TB	Teflon	48.0	4.9	7.6	13.9	42.0	10.6	48.4	
	out	TB	Teflon	44.5	4.6	8.2	13.9	42.0	10.6	48.4	
	out	TB	Sulfinert	41.0	4.2	1.1	13.9	42.0	10.6	48.4	
	out	TB	Sulfinert	46.4	4.8	1.0	13.9	42.0	10.6	48.4	
	in	TB	none	42.6	4.4	-	13.9	42.0			
	in	TB	none	44.8	4.6	-	13.9	42.0			
	in	RAD	-	-	-	-	13.9	42.0			
7 March 2022–14 March 2022	in	TB	none	47.6	4.9	-	11.2	54.6			
	in	TB	none	45.6	4.6	-	11.2	54.6			
	in	TB	none	44.8	4.6	-	11.2	54.6			
	in	RAD	-	-	-	-	13.9	42.0			
14 March 2022–21 March 2022	in	TB	none	49.8	4.8	-	12.2	64.1			
	in	TB	none	43.9	4.8	-	12.2	64.1			
	in	RAD	-	-	-	-	12.2	64.1			
21 March 2022–28 March 2022	out	TB	Teflon	40.3	4.4	8.6	18.0	36.4	13.8	43.1	
	out	TB	Sulfinert	45.6	4.7	1.0	18.0	36.4	13.8	43.1	
	in	TB	none	42.6	4.4	-	18.0	36.4			
	in	RAD	-	-	-	-	13.9	42.0			
28 March 2022–4 April 2022	in	RAD	-	-	-	-	13.9	42.0			
4 April 2022–11 April 2022	in	RAD	-	-	-	-	13.9	42.0			
11 April 2022–19 April 2022	in	RAD	-	-	-	-	13.9	42.0			
19 April 2022–25 April 2022	in	RAD	-	-	-	-	11.2	54.6			
	in	TB	none	41.0	5.0	-	16.5	50.9			First of two tubes
	in	TB	none	41.0	5.0	-	16.5	50.9			Second of two tubes
	in	Tenax	none	42.0	5.1	-	16.5	50.9			First of two tubes
	in	Tenax	none	42.0	5.1	-	16.5	50.9			Second of two tubes
2 weeks											
7 March 2022–21 March 2022	out	TB	Teflon	85.6	4.3	8.9	11.7	59.3	11.5	59.7	
	out	TB	Sulfinert	90.3	4.5	1.0	11.7	59.3	11.5	59.7	
28 March 2022–11 April 2022	in	TB	none	81.5	4.1	-	16.5	50.9			
	in	TB	none	93.0	4.7	-	16.5	50.9			
11 April 2022–25 April 2022	in	TB	none	95.2	4.7	-	17.8	46.3			
	in	TB	none	83.4	4.1	-	17.8	46.3			
4 weeks											
28 March 2022–25 April 2022	in	TB	none	199.0	4.9	-	16.9	45.2			
	in	TB	none	174.8	4.4	-	16.9	45.2			
	in	TB	none	189.6	4.8	-	16.9	45.2			
6 weeks											
14 March 2022–25 April 2022	in	TB	none	273.8	4.5	-	16.3	47.0			

## Data Availability

All data is provided in the article.

## Supplementary Materials

The following supporting information can be downloaded at: https://www.mdpi.com/article/10.3390/molecules28135001/s1, Figure S1: Stability of generated concentrations of NMHCs in breakthrough studies. In most cases each point represents an average of 24 measurements. Sometimes an average of 5 to 23 measurements is taken depending on availability of analytical data; Figure S2: Breakthrough curves for NMHCs at the concentrations of 1 and 70 µg m^−3^ sampled at a flow rate ranging from 2.5 to 4.0 mL min^−1^. Green round symbols represent the masses measured in the tubes by GC-MS analysis. Blue triangles represent theoretical doped mass values; Figure S3: Relationship between measured and theoretical values for heptane, toluene, octane, ethylbenzene, m,p-xylene, p-xylene, decane and naphthalene. In orange (barely visible) are the values from breakthrough experiments done at 1 µg m^−3^, in blue are the values from breakthrough experiments done at 10 µg m^−3^, in green are the values from breakthrough experiments done at 70 µg m^−3^. A black dashed line is a linear fit through the points obtained while doping the tubes with 1, 10 and 70 µg m^−3^. A dashed red line represents a 1:1 line; Figure S4: Profiles of heptane, toluene, octane, ethylbenzene, m,p-xylene, p-xylene, decane and naphthalene concentrations calculated using masses measured by portable GC analyzer. Measurements are taken continuously every hour from 1 of February 2021 to 27 of April 2021; Figure S5: Comparison of average weekly, by-weekly, 4-week and 6-week concentrations for (A) heptane, (B) octane, (C) decane, (D) toluene, (E) ethylbenzene, (F) m,p-xylene, (G) o-xylene, (H) naphthalene. Red error bars represent the results that are not in accordance with the measurements obtained from portable GC; Table S1: Select NMHCs sampling rates for RAD 145 sampling device when sampled for 1 week at 25 °C.
