# Supplementary material for: Development of a Long-Term Sampling Method for Determination of NMHCs in Indoor Air
Source: Molecules. 2023 Jun 26;28(13):5001. doi: 10.3390/molecules28135001 (PMC10343520; doi:10.3390/molecules28135001)
Supplement: Supplementary file 1 [file molecules-28-05001-s001.zip › molecules-2462905-supplementary.pdf]

Supplementary Material for:

Development of a Long-Term Sampling Method for Determination of NMHCs in Indoor Air

Darya Urupina [1,*], Sylvie Traverse [2], Thierry Leonardis [1], Elise Eymard-Vernain [3], Julien Guilhermet [3], Vincent Ricard [3], Marie Lemoine [2], Camille Varlet [1], Remy Gillet [2] and Nadine Locoge [1]

[1] IMT Nord Europe, Institut Mines-Telecom, Centre for Energy and Environment, University Lille, 59000 Lille, France; thierry.leonardis@imt-nord-europe.fr (T.L.); camille.varlet@imt-nord-europe.fr (C.V.); nadine.locoge@imt-nord-europe.fr (N.L.)

[2] GINGER-BURGEAP, 19 Rue de la Villette, CEDEX 3, 69425 Lyon, France; s.traverse@groupeginger.com (S.T.); m.lemoine@groupeginger.com (M.L.); r.gillet@groupeginger.com (R.G.)

[3] TERA Environment, 628 Rue Charles de Gaulle, 38920 Crolles, France; elise.eymardvernain@tera-environnement.com (E.E.-V.); julien.guilhermet@tera-environnement.com (J.G.); vincent.ricard@tera-environnement.com (V.R.)

* Correspondence: darya.urupina@imt-nord-europe.fr

Tables

Table S1: Select NMHCs sampling rates for RAD 145 sampling device when sampled for 1 week at 25 °C.

analyte	Sampling rates (ml.min^{-1})	Exposure time upper limit (days)
hexane	25.5	7
benzene	27.8	7
heptane	25.3	14
toluene	30.0	14
octane	24.1	14
ethylbenzene	25.7	14
meta + para-xylene	26.6	14
o-xylene	24.6	14
decane	22.3	14
naphthalene	20*	-

* The value is not provided by Radiello guide and is calculated by Maugeri foundation.

Figures

Figure S1: Stability of generated concentrations of NMHCs in breakthrough studies. In most cases each point represents an average of 24 measurements. Sometimes an average of 5 to 23 measurements is taken depending on availability of analytical data.

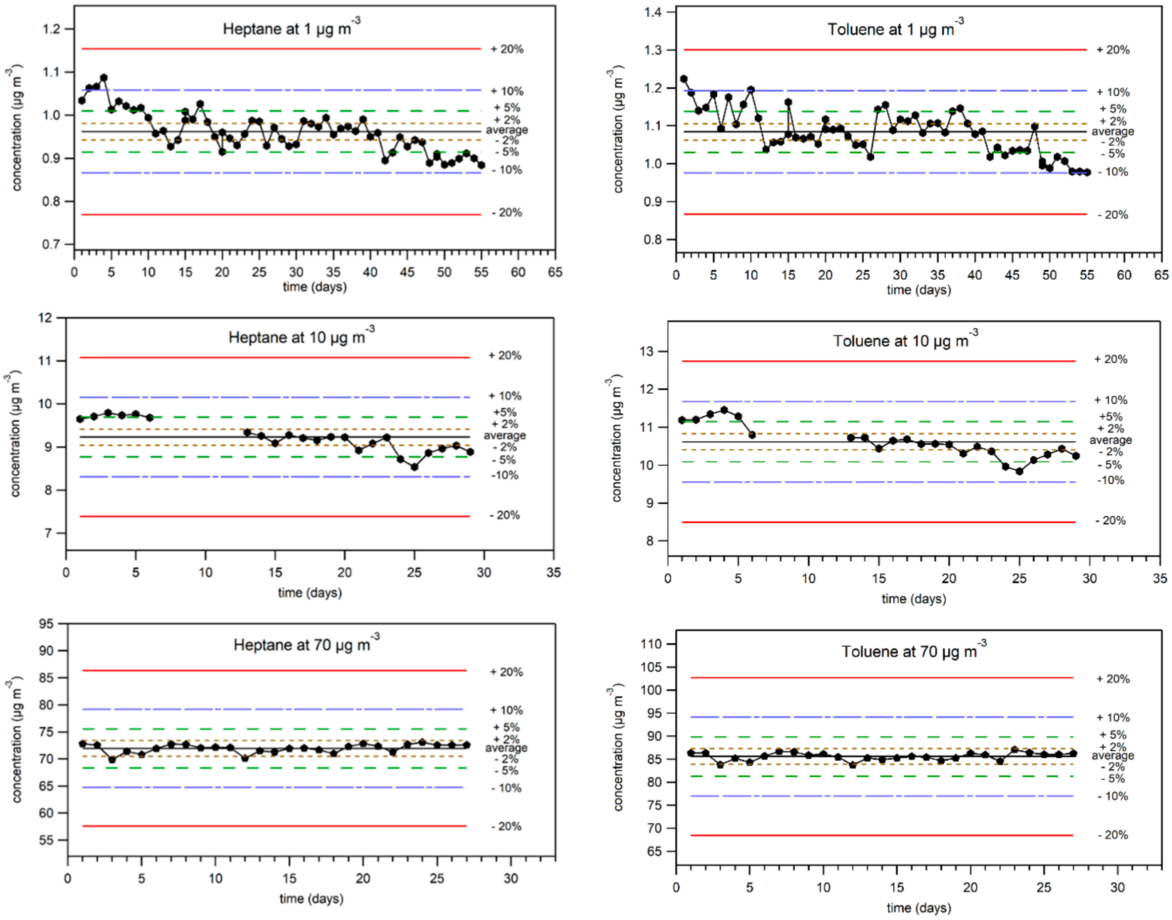

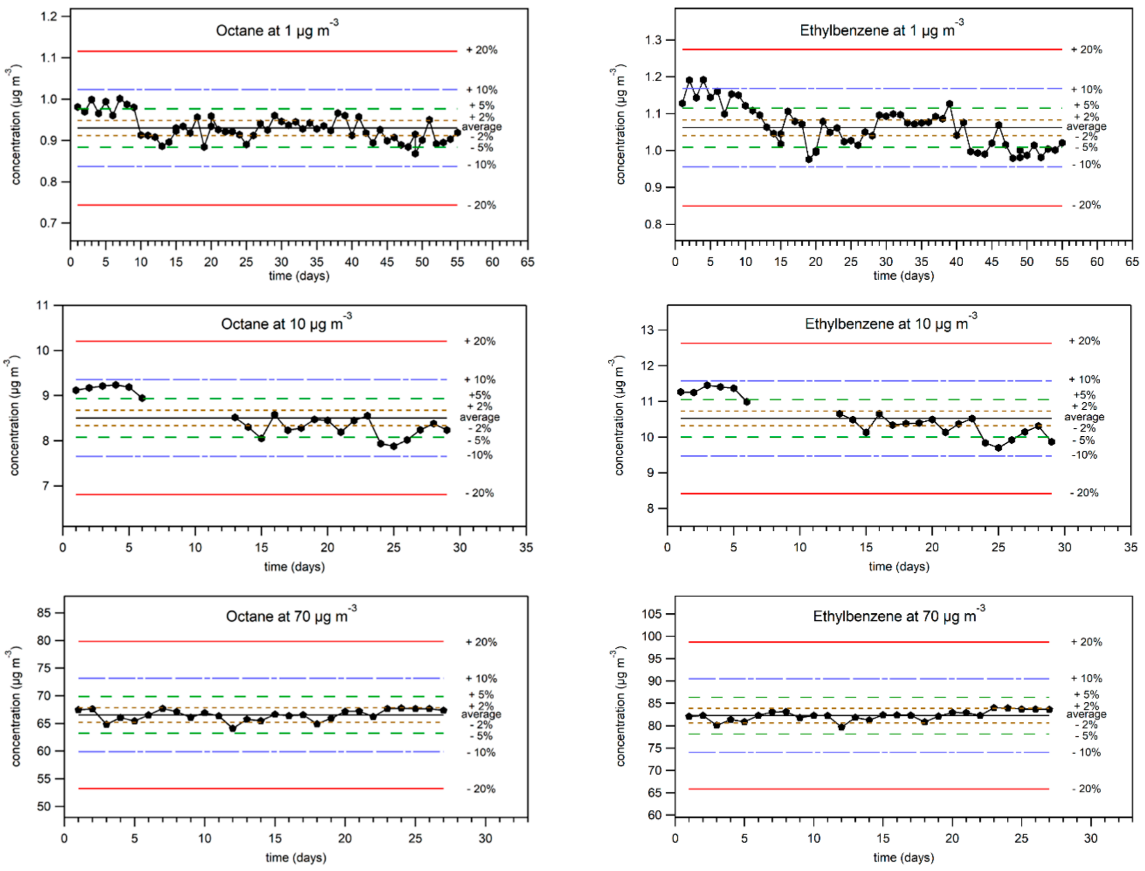
Octane at 1 µg m-3
Ethylbenzene at 1 µg m-3
Octane at 10 µg m-3
Ethylbenzene at 10 µg m-3
Octane at 70 µg m-3
Ethylbenzene at 70 µg m-3
concentration (µg m-3)
time (days)
+ 20%
+ 10%
+ 5%
+ 2%
average
- 2%
- 5%
- 10%
- 20%

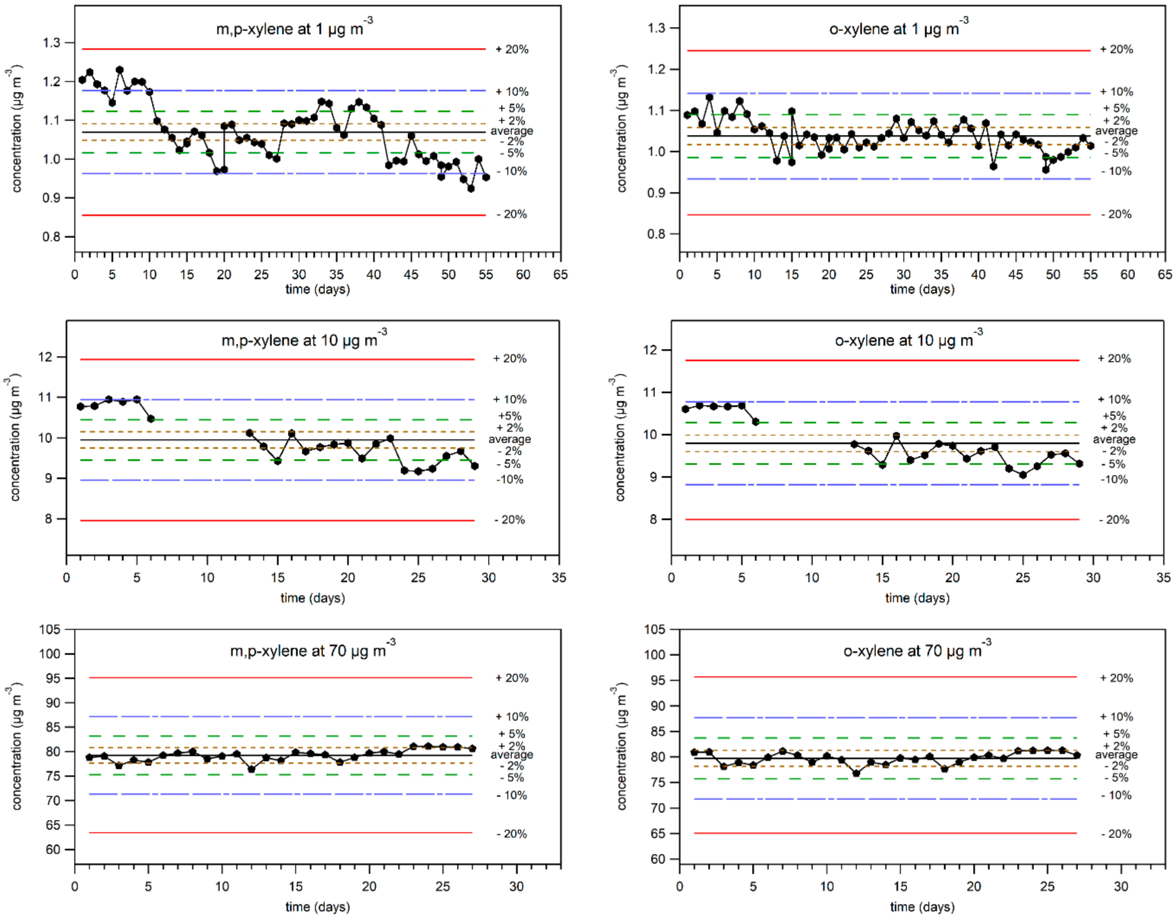
m,p-xylene at 1 µg m-3
o-xylene at 1 µg m-3
m,p-xylene at 10 µg m-3
o-xylene at 10 µg m-3
m,p-xylene at 70 µg m-3
o-xylene at 70 µg m-3
concentration (µg m-3)
time (days)
+ 20%
+ 10%
+ 5%
+ 2%
average
- 2%
- 5%
- 10%
- 20%

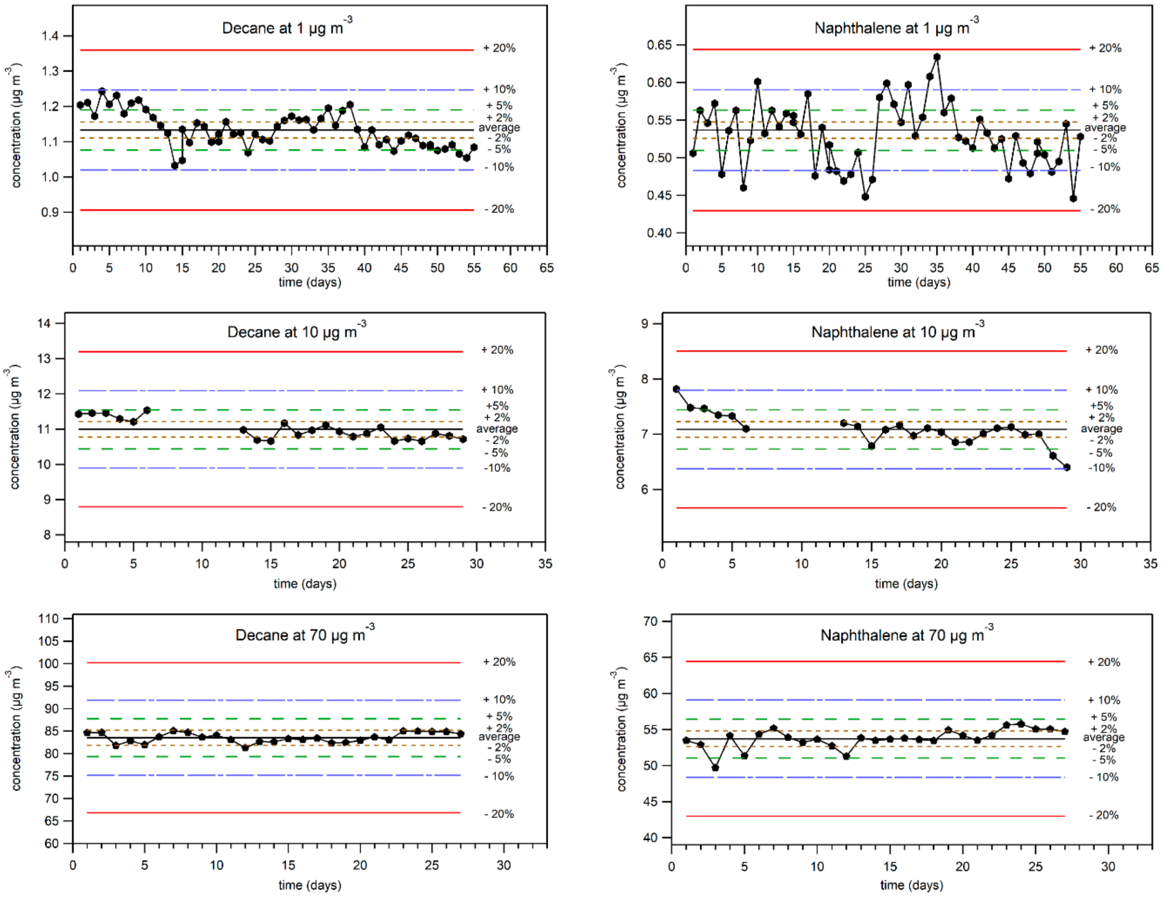
Decane at 1 µg m-3
Naphthalene at 1 µg m-3
Decane at 10 µg m-3
Naphthalene at 10 µg m-3
Decane at 70 µg m-3
Naphthalene at 70 µg m-3
concentration (µg m-3)
time (days)
+ 20%
+ 10%
+ 5%
+ 2%
average
- 2%
- 5%
- 10%
- 20%

Figure S2: Breakthrough curves for NMHCs at the concentrations of 1 and 70 µg m^{-3} sampled at a flow rate ranging from 2.5 to 4.0 ml min^{-1}. Green round symbols represent the masses measured in the tubes by GC-MS analysis. Blue triangles represent theoretical doped mass values.

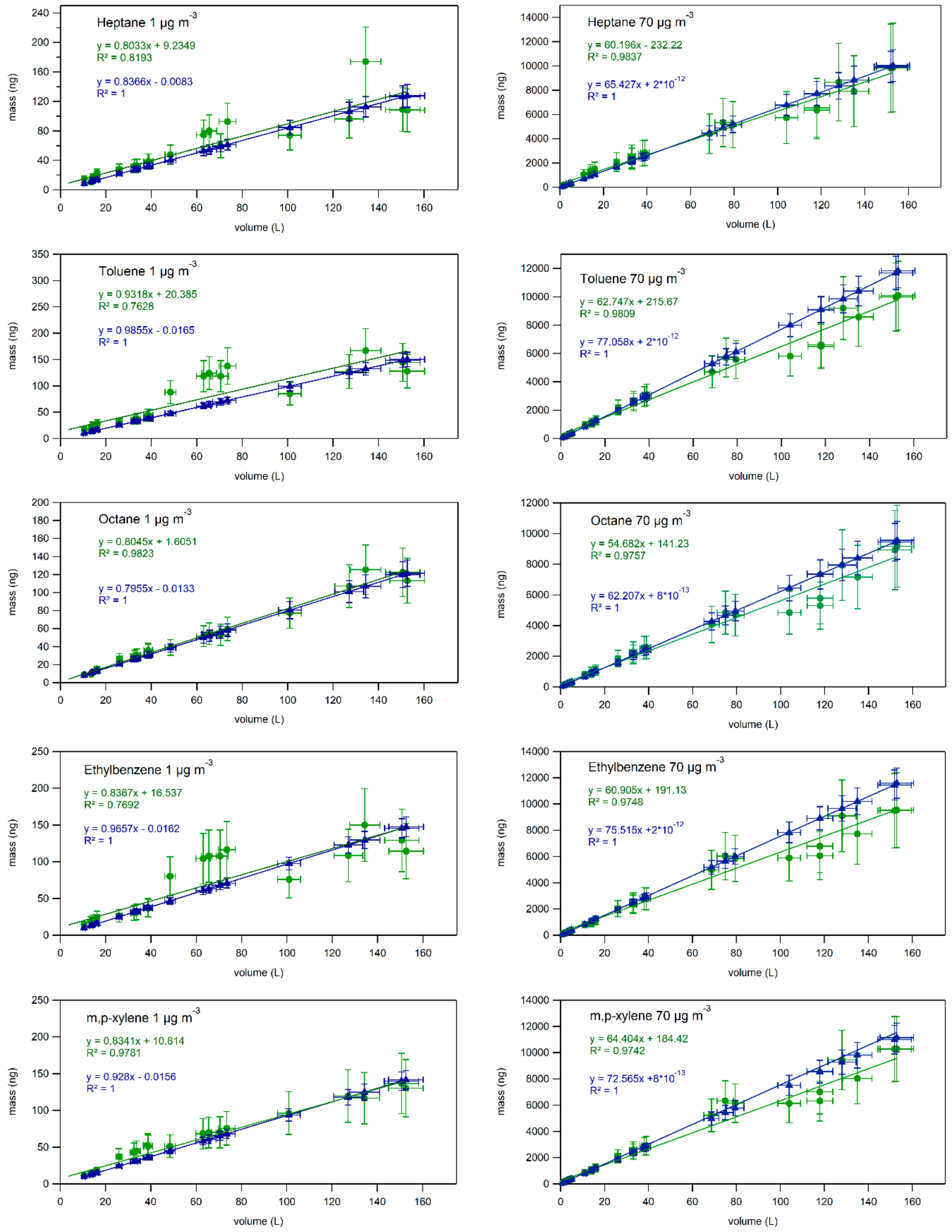

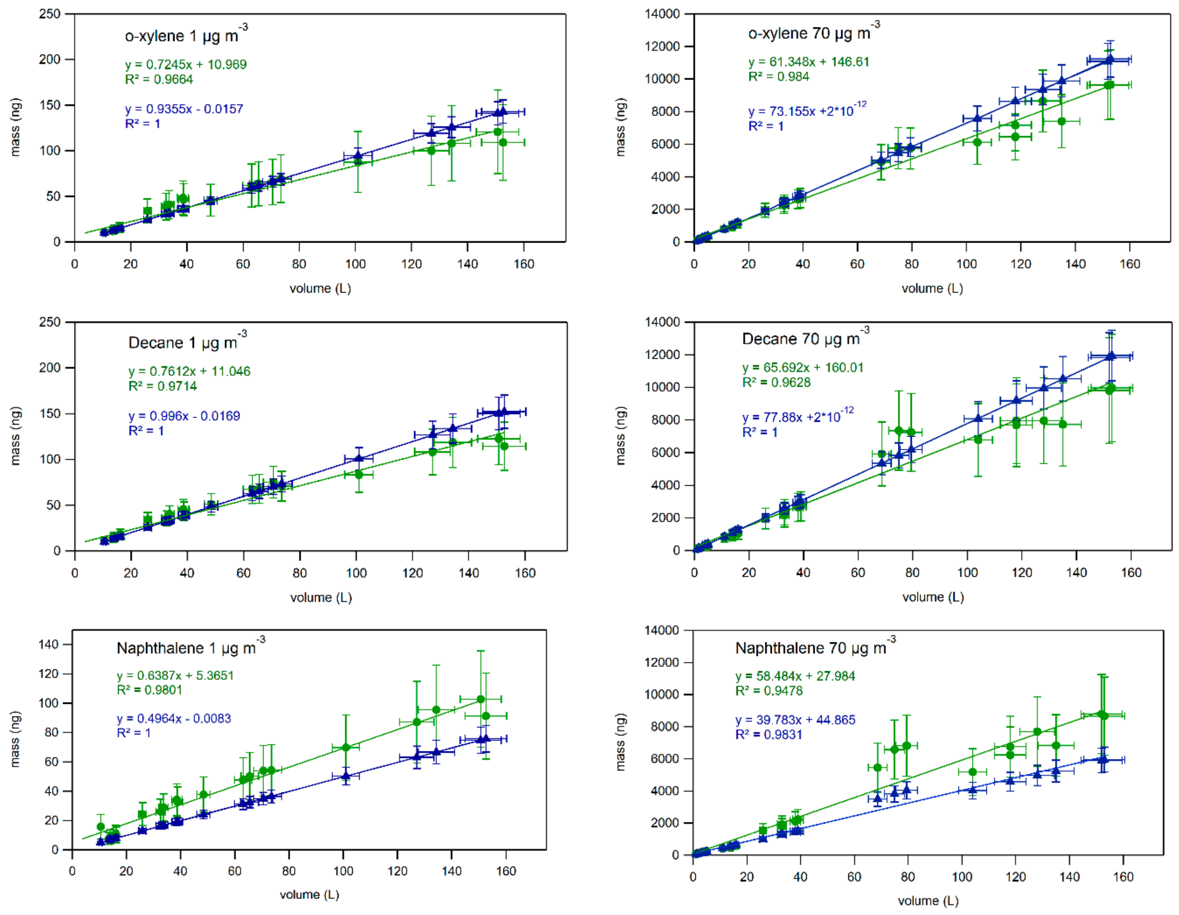
o-xylene 1 µg m-3
y = 0.7245x + 10.969
R² = 0.9664
y = 0.9355x - 0.0157
R² = 1
o-xylene 70 µg m-3
y = 61.348x + 146.61
R² = 0.984
y = 73.155x +2*10-12
R² = 1
Decane 1 µg m-3
y = 0.7612x + 11.046
R² = 0.9714
y = 0.996x - 0.0169
R² = 1
Decane 70 µg m-3
y = 65.692x + 160.01
R² = 0.9628
y = 77.88x +2*10-12
R² = 1
Naphthalene 1 µg m-3
y = 0.6387x + 5.3651
R² = 0.9801
y = 0.4964x - 0.0083
R² = 1
Naphthalene 70 µg m-3
y = 58.484x + 27.984
R² = 0.9478
y = 39.783x + 44.865
R² = 0.9831
mass (ng)
volume (L)

Figure S3: Relationship between measured and theoretical values for heptane, toluene, octane, ethylbenzene, m,p-xylene, p-xylene, decane and naphthalene. In orange (barely visible) are the values from breakthrough experiments done at 1 µg m^{-3}, in blue are the values from breakthrough experiments done at 10 µg m^{-3}, in green are the values from breakthrough experiments done at 70 µg m^{-3}. A black dashed line is a linear fit through the points obtained while doping the tubes with 1, 10 and 70 µg m^{-3}. A dashed red line represents a 1:1 line.

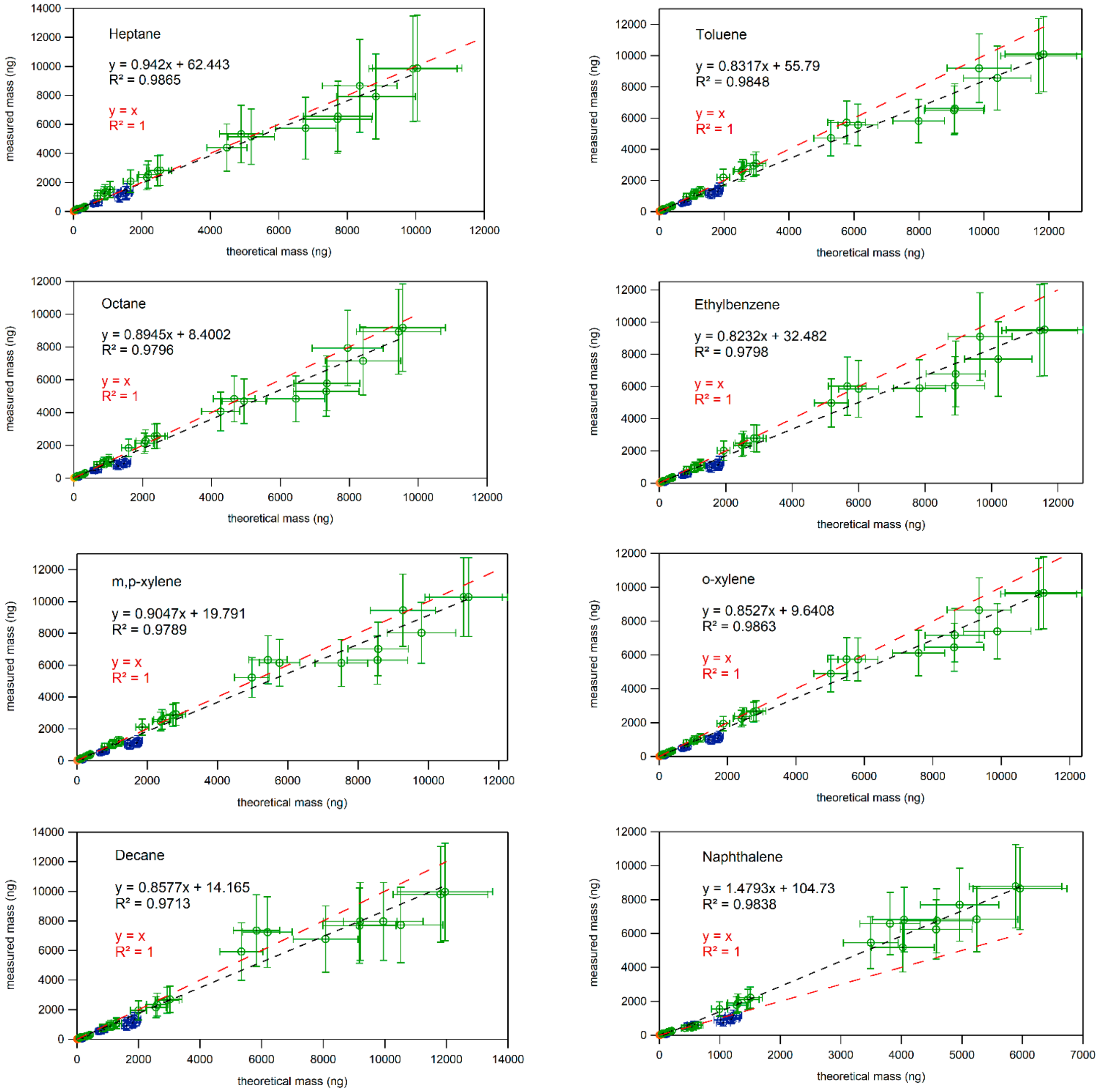

Figure S4: Profiles of heptane, toluene, octane, ethylbenzene, m,p-xylene, p-xylene, decane and naphthalene concentrations calculated using masses measured by portable GC analyzer. Measurements are taken continuously every hour from 1 of February 2021 to 27 of April 2021.

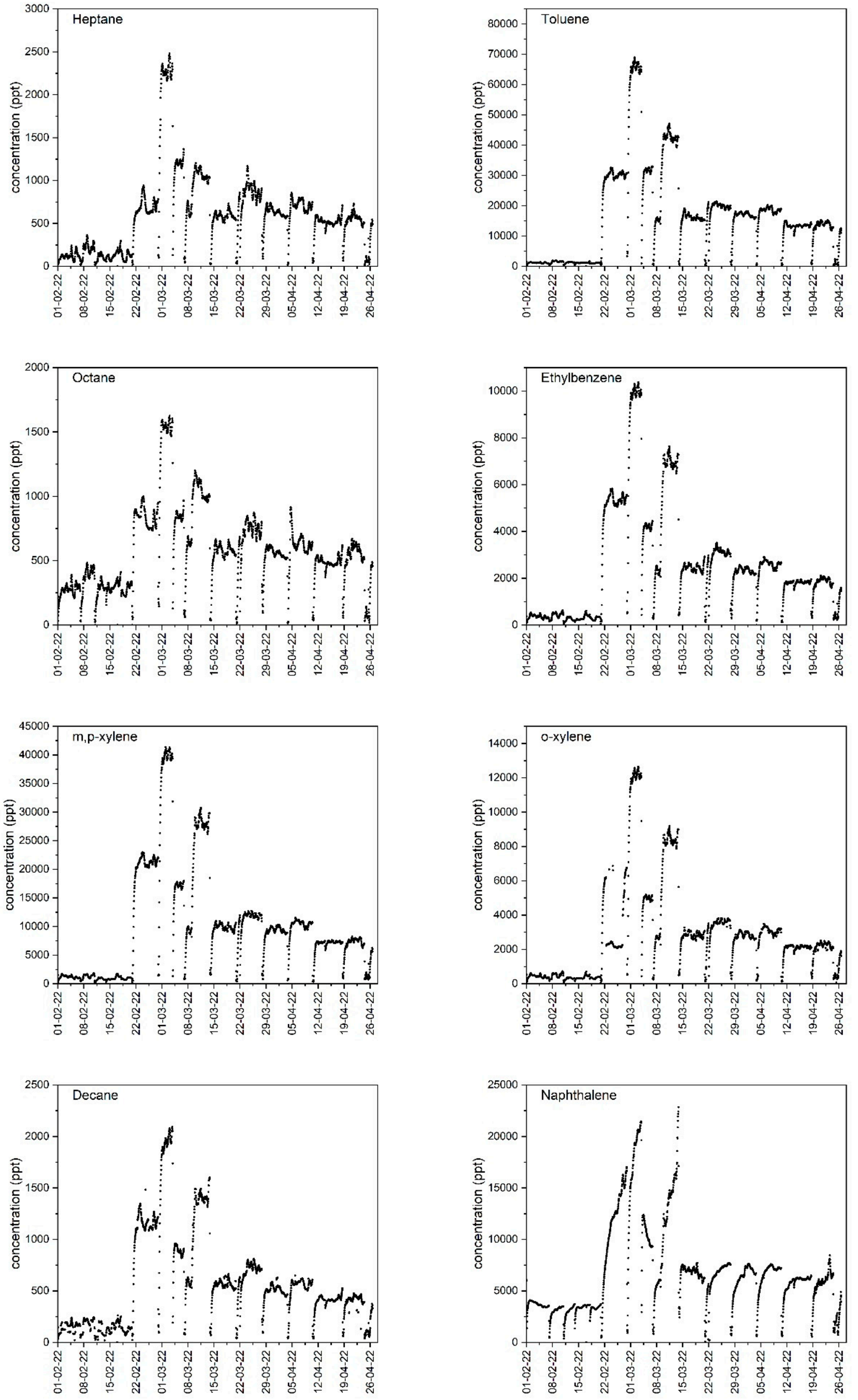

Figure S5: Comparison of average weekly, by-weekly, 4-week and 6-week concentrations for A) heptane, B) octane, C) decane, D) toluene, E) ethylbenzene, F) m,p-xylene, G) o-xylene, H) naphthalene. Red error bars represent the results that are not in accordance with the measurements obtained from portable GC.

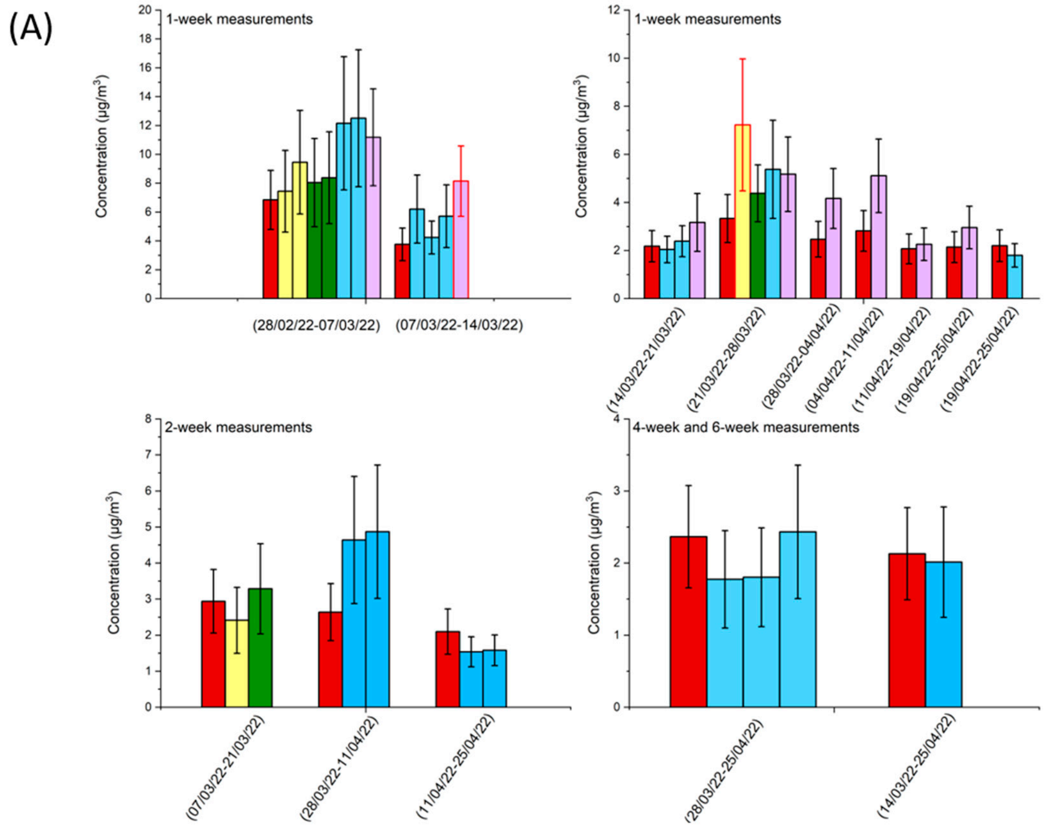

- *average concentration calculated from data obtained from portable GC*
- *concentrations measured using the TB tubes placed outside of the experimental room and connected to it using PTFE tubing*
- *concentrations measured using the TB tubes placed outside of the experimental room and connected to it using Sulfinert tubing*
- *concentrations obtained from the TB tubes put directly inside of the testing room*
- *concentrations measured weekly by RAD samplers placed directly in the testing room*

(B)

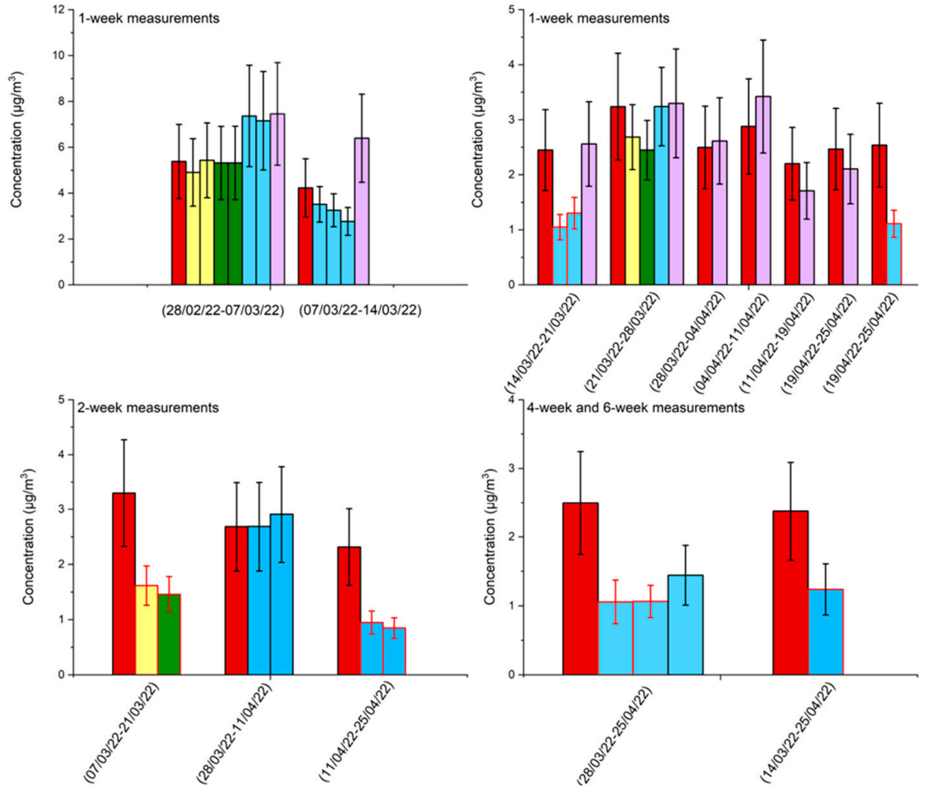
1-week measurements
Concentration (μg/m³)
(28/02/22-07/03/22)
(07/03/22-14/03/22)
1-week measurements
Concentration (μg/m³)
(14/03/22-21/03/22)
(21/03/22-28/03/22)
(28/03/22-04/04/22)
(04/04/22-11/04/22)
(11/04/22-19/04/22)
(19/04/22-25/04/22)
(19/04/22-25/04/22)
2-week measurements
Concentration (μg/m³)
(07/03/22-21/03/22)
(28/03/22-11/04/22)
(11/04/22-25/04/22)
4-week and 6-week measurements
Concentration (μg/m³)
(28/03/22-25/04/22)
(14/03/22-25/04/22)

(C)

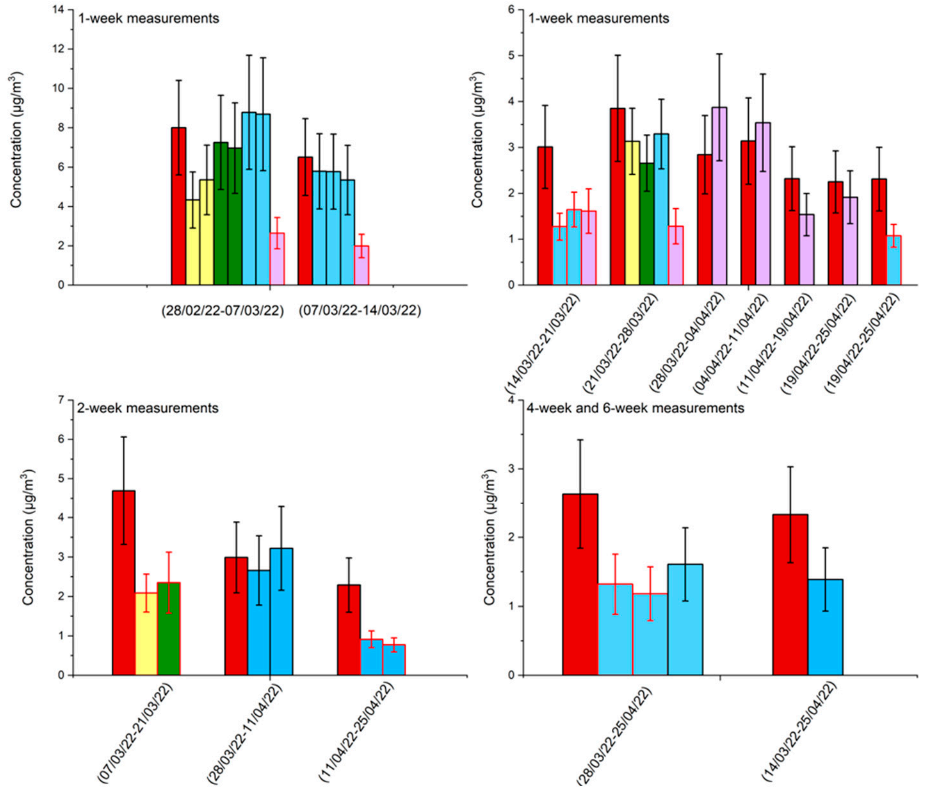
1-week measurements
Concentration (μg/m³)
(28/02/22-07/03/22)
(07/03/22-14/03/22)
1-week measurements
Concentration (μg/m³)
(14/03/22-21/03/22)
(21/03/22-28/03/22)
(28/03/22-04/04/22)
(04/04/22-11/04/22)
(11/04/22-19/04/22)
(19/04/22-25/04/22)
(19/04/22-25/04/22)
2-week measurements
Concentration (μg/m³)
(07/03/22-21/03/22)
(28/03/22-11/04/22)
(11/04/22-25/04/22)
4-week and 6-week measurements
Concentration (μg/m³)
(28/03/22-25/04/22)
(14/03/22-25/04/22)

(D)

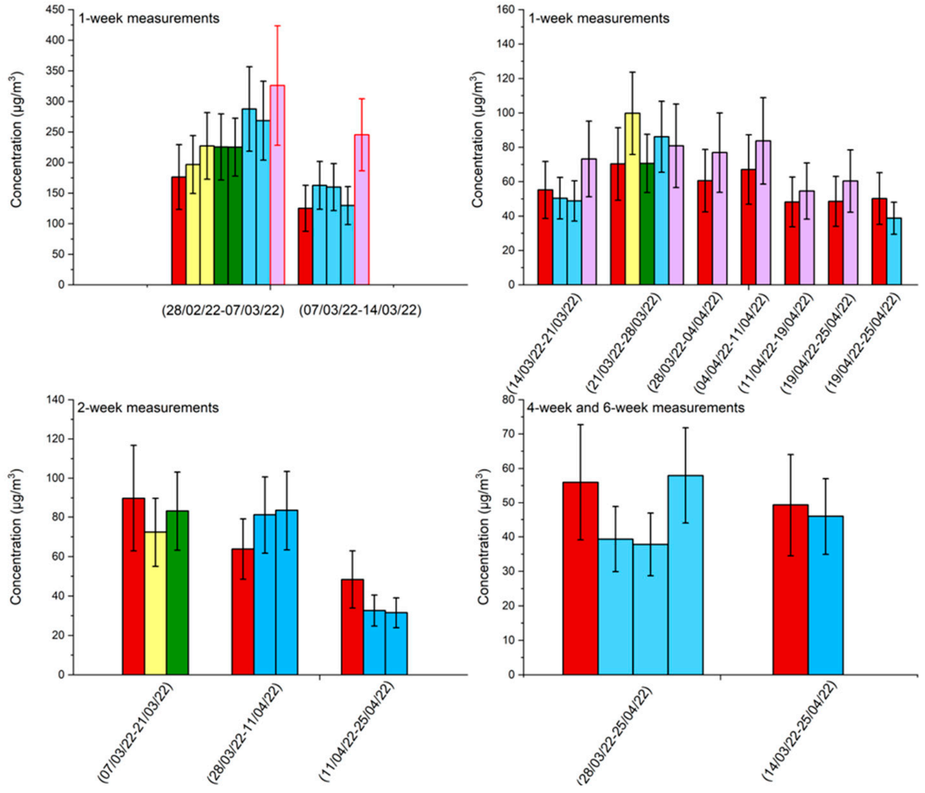
1-week measurements
Concentration (µg/m3)
(28/02/22-07/03/22)
(07/03/22-14/03/22)
1-week measurements
(14/03/22-21/03/22)
(21/03/22-28/03/22)
(28/03/22-04/04/22)
(04/04/22-11/04/22)
(11/04/22-19/04/22)
(19/04/22-25/04/22)
(19/04/22-25/04/22)
2-week measurements
(07/03/22-21/03/22)
(28/03/22-11/04/22)
(11/04/22-25/04/22)
4-week and 6-week measurements
(28/03/22-25/04/22)
(14/03/22-25/04/22)

(E)

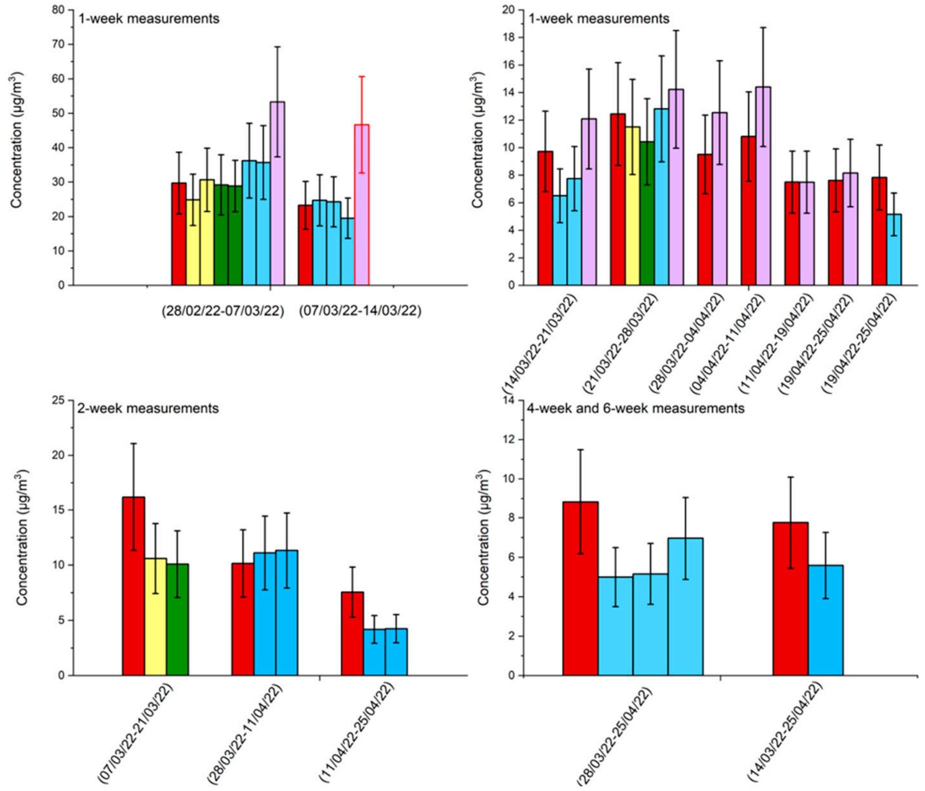
1-week measurements
Concentration (µg/m3)
(28/02/22-07/03/22)
(07/03/22-14/03/22)
1-week measurements
(14/03/22-21/03/22)
(21/03/22-28/03/22)
(28/03/22-04/04/22)
(04/04/22-11/04/22)
(11/04/22-19/04/22)
(19/04/22-25/04/22)
(19/04/22-25/04/22)
2-week measurements
(07/03/22-21/03/22)
(28/03/22-11/04/22)
(11/04/22-25/04/22)
4-week and 6-week measurements
(28/03/22-25/04/22)
(14/03/22-25/04/22)

(F)

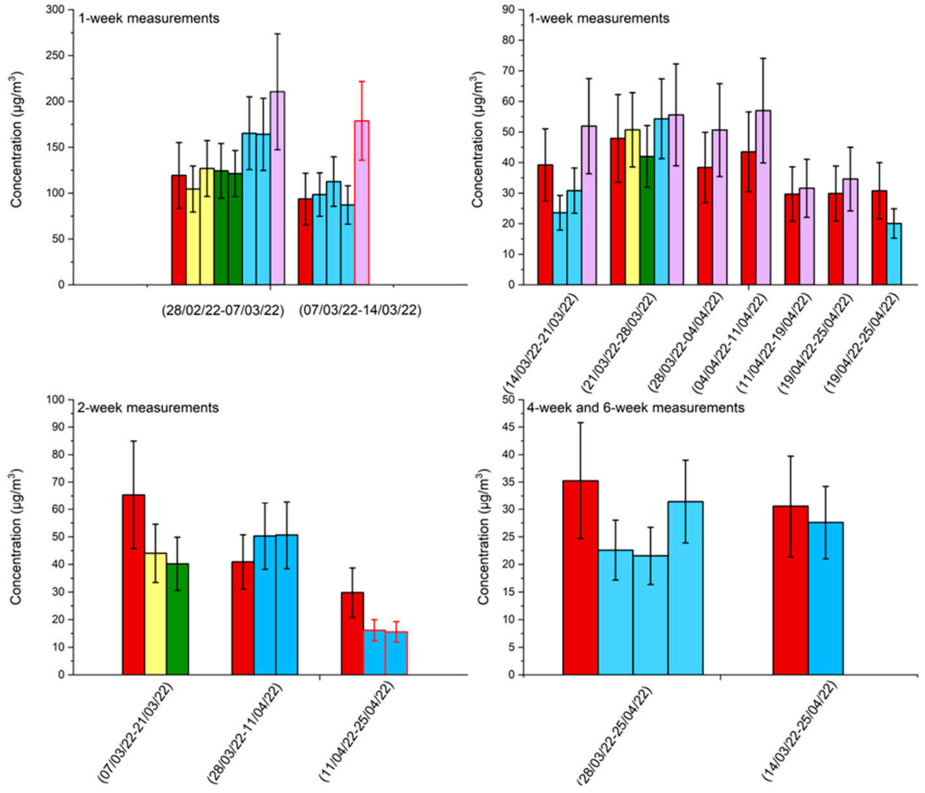
1-week measurements
Concentration (µg/m3)
(28/02/22-07/03/22)
(07/03/22-14/03/22)
1-week measurements
Concentration (µg/m3)
(14/03/22-21/03/22)
(21/03/22-28/03/22)
(28/03/22-04/04/22)
(04/04/22-11/04/22)
(11/04/22-19/04/22)
(19/04/22-25/04/22)
(19/04/22-25/04/22)
2-week measurements
Concentration (µg/m3)
(07/03/22-21/03/22)
(28/03/22-11/04/22)
(11/04/22-25/04/22)
4-week and 6-week measurements
Concentration (µg/m3)
(28/03/22-25/04/22)
(14/03/22-25/04/22)

(G)

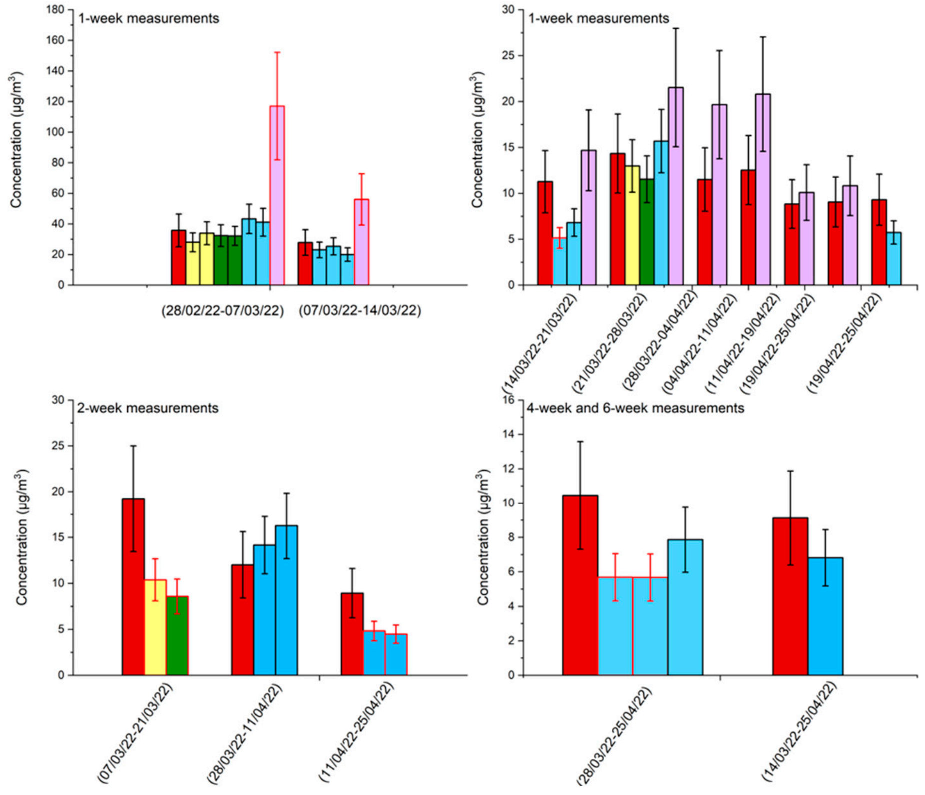
1-week measurements
Concentration (µg/m3)
(28/02/22-07/03/22)
(07/03/22-14/03/22)
1-week measurements
Concentration (µg/m3)
(14/03/22-21/03/22)
(21/03/22-28/03/22)
(28/03/22-04/04/22)
(04/04/22-11/04/22)
(11/04/22-19/04/22)
(19/04/22-25/04/22)
(19/04/22-25/04/22)
2-week measurements
Concentration (µg/m3)
(07/03/22-21/03/22)
(28/03/22-11/04/22)
(11/04/22-25/04/22)
4-week and 6-week measurements
Concentration (µg/m3)
(28/03/22-25/04/22)
(14/03/22-25/04/22)

(H)

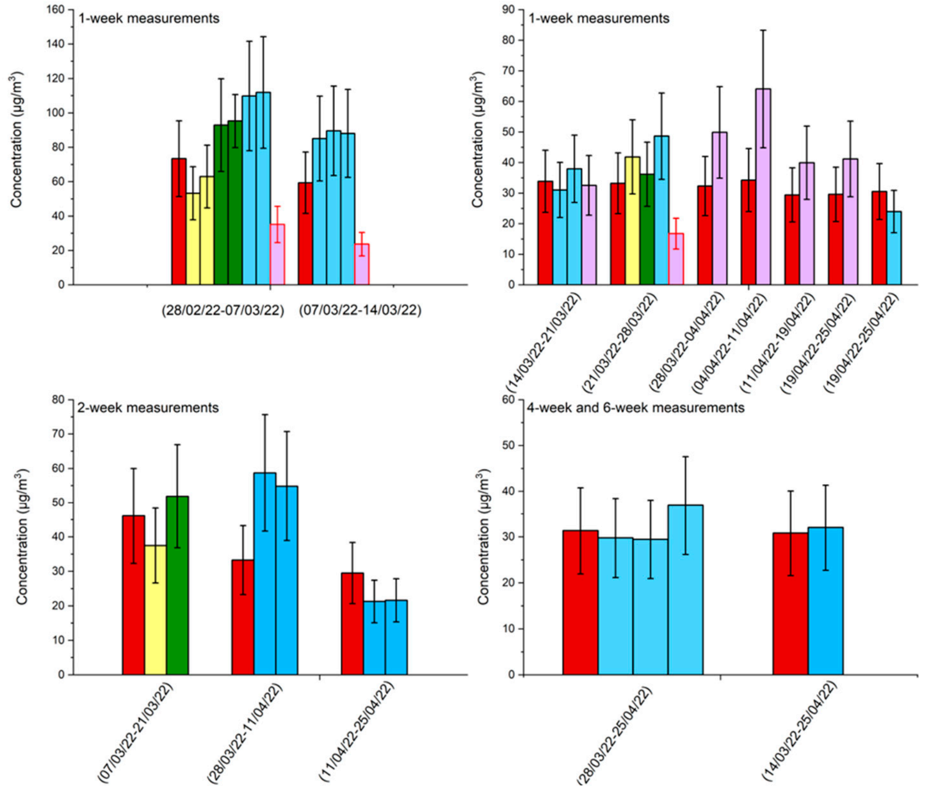
1-week measurements
Concentration (μg/m³)
(28/02/22-07/03/22)
(07/03/22-14/03/22)
1-week measurements
(14/03/22-21/03/22)
(21/03/22-28/03/22)
(28/03/22-04/04/22)
(04/04/22-11/04/22)
(11/04/22-19/04/22)
(19/04/22-25/04/22)
(19/04/22-25/04/22)
2-week measurements
(07/03/22-21/03/22)
(28/03/22-11/04/22)
(11/04/22-25/04/22)
4-week and 6-week measurements
(28/03/22-25/04/22)
(14/03/22-25/04/22)
